# HMGA1 promotes breast cancer angiogenesis supporting the stability, nuclear localization and transcriptional activity of FOXM1

**DOI:** 10.1186/s13046-019-1307-8

**Published:** 2019-07-16

**Authors:** Rossella Zanin, Silvia Pegoraro, Gloria Ros, Yari Ciani, Silvano Piazza, Fleur Bossi, Roberta Bulla, Cristina Zennaro, Federica Tonon, Dejan Lazarevic, Elia Stupka, Riccardo Sgarra, Guidalberto Manfioletti

**Affiliations:** 1 Department of Life Sciences, University of Trieste, 34127 Trieste, Italy; 2 Laboratorio Nazionale CIB, Area Science Park, Padriciano 99, Trieste, Italy; 3 Department of Cellular, Computational and Integrative Biology - (CIBIO), University of Trento, Via Sommarive 9, 38123, Povo, Trento, Italy; 4 Institute for Maternal and Child Health, Istituto di Ricovero e Cura a Carattere Scientifico (I.R.C.C.S.) “Burlo Garofolo”, via dell’Istria 65/1, 34134 Trieste, Italy; 5 Department of Medicine, Surgery and Health Sciences, University of Trieste, 34149 Trieste, Italy; 6 Center for Translational Genomics and Bioinformatics, IRCCS San Raffaele Scientific Institute, Milan, Italy; 7 Present address: Department of Cellular, Computational and Integrative Biology - (CIBIO), University of Trento, Via Sommarive 9, 38123 Trento, Italy; 8 Present address: Life Sciences Business Health Catalyst, Cambridge, Via Sommarive 9, 38123 USA

**Keywords:** HMGA1, FOXM1, Triple-negative breast cancer, Gene network, VEGFA, Angiogenesis

## Abstract

**Background:**

Breast cancer is the most common malignancy in women worldwide. Among the breast cancer subtypes, triple-negative breast cancer (TNBC) is the most aggressive and the most difficult to treat. One of the master regulators in TNBC progression is the architectural transcription factor HMGA1. This study aimed to further explore the HMGA1 molecular network to identify molecular mechanisms involved in TNBC progression.

**Methods:**

RNA from the MDA-MB-231 cell line, silenced for HMGA1 expression, was sequenced and, with a bioinformatic analysis, molecular partners HMGA1 could cooperate with in regulating common downstream gene networks were identified. Among the putative partners, the FOXM1 transcription factor was selected. The relationship occurring between HMGA1 and FOXM1 was explored by qRT-PCR, co-immunoprecipitation and protein stability assays. Subsequently, the transcriptional activity of HMGA1 and FOXM1 was analysed by luciferase assay on the VEGFA promoter. The impact on angiogenesis was assessed in vitro, evaluating the tube formation ability of endothelial cells exposed to the conditioned medium of MDA-MB-231 cells silenced for HMGA1 and FOXM1 and in vivo injecting MDA-MB-231 cells, silenced for the two factors, in zebrafish larvae.

**Results:**

Here, we discover FOXM1 as a novel molecular partner of HMGA1 in regulating a gene network implicated in several breast cancer hallmarks. HMGA1 forms a complex with FOXM1 and stabilizes it in the nucleus, increasing its transcriptional activity on common target genes, among them, VEGFA, the main inducer of angiogenesis. Furthermore, we demonstrate that HMGA1 and FOXM1 synergistically drive breast cancer cells to promote tumor angiogenesis both in vitro in endothelial cells and in vivo in a zebrafish xenograft model. Moreover, using a dataset of breast cancer patients we show that the co-expression of HMGA1, FOXM1 and VEGFA is a negative prognostic factor of distant metastasis-free survival and relapse-free survival.

**Conclusions:**

This study reveals FOXM1 as a crucial interactor of HMGA1 and proves that their cooperative action supports breast cancer aggressiveness, by promoting tumor angiogenesis. Therefore, the possibility to target HMGA1/FOXM1 in combination should represent an attractive therapeutic option to counteract breast cancer angiogenesis.

**Electronic supplementary material:**

The online version of this article (10.1186/s13046-019-1307-8) contains supplementary material, which is available to authorized users.

## Background

Breast cancer (BC) is the most commonly diagnosed cancer and is the leading cause of cancer-related death in women. Different breast cancer subtypes have been described based on gene expression analysis and it is widely accepted that there are five distinct intrinsic molecular subtypes: luminal A, luminal B, HER2-enriched, normal-like and basal-like breast cancer [1]. The majority of basal-like breast cancers are defined triple-negative breast cancers (TNBC), as they lack the expression of the estrogen receptor (ER), progesterone receptor (PR) and human epidermal growth factor receptor 2 (HER2/neu), making them difficult to treat [2, 3]. TNBC accounts for approximately 15% of invasive breast cancers and represents the most aggressive breast cancer subtype: indeed TNBC is a typically high-grade tumor, poorly differentiated, and associated with poor prognosis and molecular heterogeneity [2–4]. Since TNBC does not respond to hormonal and target therapies, the only current therapeutic options are represented by tumor excision, radiation therapy and conventional chemotherapy. Increasing evidence points out that the microvascular density in TNBC is higher with respect to other breast cancers subtypes, thus highlighting that angiogenesis crucially supports TNBC progression. Therefore, the study of efficacious anti-angiogenic therapies in TNBC is critical [5, 6].

One of the factors involved in TNBC aggressiveness is the High Mobility Group A1 (HMGA1), a member of non-histone chromatin proteins [7]. HMGA1 is an architectural transcription factor which, by altering chromatin structure and interacting with transcription factors, can regulate the transcription of several genes [8–10]. HMGA1 is defined as an oncofetal protein due to its expression pattern: indeed, it is highly expressed during embryogenesis, while its expression decreases or is absent in adults; it is then re-expressed in a variety of tumors, including breast cancer [11, 12]. In this context, several works established that HMGA1 expression is correlated with high tumor grade and metastatization, resistance to therapies and poor prognosis [7, 13–15]. Furthermore, a causal role of HMGA1 in breast cancer onset and progression has been demonstrated. In fact, HMGA1 over-expression in non-tumorigenic MCF-7 human breast epithelial cells leads to the acquisition of a transformed and aggressive phenotype [16], whereas HMGA1 silencing in highly aggressive TNBC cell lines causes the reversion of the tumorigenic phenotype, as assessed both by in vitro and in vivo approaches [13, 17]. We have previously reported that HMGA1 exerts its action by governing the transcription of gene networks fundamental in supporting TNBC aggressiveness [13, 18–20]. In detail, HMGA1 induces the expression of epithelial to mesenchymal transition (EMT) - and stemness-associated genes and, through the regulation of CCNE2/CDK2 complex, it is able to modulate the Hippo pathway, finally regulating the localization and activity of YAP and conferring metastatic abilities to TNBC cells [18]. In addition, HMGA1 regulates a gene network linked to secreted proteins. In fact, by inducing the expression of key components of the plasminogen activation system, such as PLAU and SERPINE1, HMGA1 is able to modulate the TNBC cell secretome stimulating TNBC cell migration in an autocrine way [20]. However, little is known about how HMGA1 governs these cancer-related gene networks.

In order to identify HMGA1-molecular partners involved in developing TNBC aggressiveness, we performed RNA sequencing analysis (RNA-Seq) on the MDA-MB-231 TNBC cell line at 24 and 72 h after HMGA1 silencing, looking for transcription factors as putative upstream regulators of HMGA1-gene networks. We found Forkhead box M1 (FOXM1) transcription factor as a novel HMGA1-molecular partner. FOXM1 overexpression is detected in a variety of human cancers, where it drives the expression of critical genes involved in the regulation of different cancer hallmarks including high proliferation, invasion, drug resistance and angiogenesis; moreover, its overexpression is associated with poor clinical prognosis [21, 22]. Intriguingly, FOXM1-associated pathway has been found to be the top up-regulated pathway in TNBC but not in other breast cancer subtypes, suggesting a crucial role of FOXM1 in TNBC [23]. Our results indicate that HMGA1 and FOXM1 together regulate a common pro-tumorigenic TNBC gene network. Specifically, HMGA1 stabilizes FOXM1 in the nucleus preventing its degradation and increasing FOXM1-dependent transcriptional activity. Furthermore, we found that HMGA1 and FOXM1 cooperatively promote the tumor angiogenic process in in vitro and in vivo models. Our study thereby describes a new molecular mechanism fundamental in TNBC aggressiveness.

## Materials and methods

### Cell culture and treatments

MDA-MB-231, MDA-MB-157 and HEK293T cell lines were routinely grown in high glucose DMEM, with 10% tetracycline-free FBS, 2 mM L-Glutamine, 100 U/ml Penicillin and 100 μg/ml Streptomycin (Euroclone). Lipofectamine™ RNAiMAX reagent (Thermo Fisher Scientific) was used for transfection of 30 pmol of siRNA/35-mm dish, following the manufacturer instructions. For co-silencing experiments, 30 pmol of each specific siRNA was used up to a final amount of 60 pmol/condition. The cells were processed after 24, 48 and 72 h of silencing, depending on the specific experiment. siCTRL and siRNAs against HMGA1 and FOXM1 have been previously used [13, 24]. Plasmid transfections were carried out using Lipofectamine 3000 (Invitrogen/ThermoFisher Scientific) for MDA-MB-231 cells following the manufacturer protocols, and the standard Calcium Phosphate transfection method for HEK293T cells. For the treatment with MG132 proteasome inhibitor (Sigma), 48 h after the siRNA transfection, MDA-MB-231 cells were treated with 10 μM of the proteasome inhibitor MG132 or DMSO, as negative control, for 6 h and then lysed in SDS sample buffer [62.5 mM Tris, pH 6.8; 2% SDS; 10% glycerol; 200 mM DTT, 1 mM Na_3_VO_4_, 5 mM NaF and mammalian protease inhibitor cocktail (PIC) (Sigma)] for Western blot analysis. For the cycloheximide (Sigma) treatment, 48 h after siRNA transfection, MDA-MB-231 cells were treated with 50 μM of cycloheximide and lysed at different time points (45, 90, 150 and 240 min) in SDS sample buffer for western blot analysis.

### Plasmids construction

pEGFP-N1, pEGFP-N1 HMGA1a, pRL-CMV Renilla (Promega) and pGL4.11 (Promega) were already present in the laboratory. pEGFP-FOXM1 and pGL3-5BS (containing five repetitions of FOXM1 binding sites-TAAACA) were kind gifts of Dr. Muy Teck Teh (Department of Diagnostic and Oral Sciences, Blizard Institute, Barts and The London School of Medicine and Dentistry, Queen Mary University of London, London, UK). The vectors used in this work named pGL4.10-VEGFprom (− 1000–1), pGL4.10-VEGFprom (− 1000–500), pGL4.10-VEGFprom (− 500–1), as they contain a portion of the VEGFA promoter that goes from − 1000 to − 1 bp, from − 1000 to − 500 bp and from − 500 to − 1 bp respectively, were a gift from David Mu (Addgene plasmid #66128, #66129, #66130). The deletion mutants pGL4.11-VEGFpromWT(− 388–1), pGL4.11-VEGFprom (− 338–1), pGL4.11-VEGFprom (− 172–1) and the pGL4.11-VEGFprom (− 104–1), containing a region of the VEGFA promoter spanning from − 388 to − 1 bp, from − 338 to − 1 bp, from − 172 to − 1 bp, and from − 104 to − 1 bp from the TSS respectively, were generated in the laboratory by amplifying the pGL4.10-VEGFprom (− 1000–1) with the forward primers 5′-GGGGTACCCCGGGGCGGGCCGGGGGCGGGGTCC-3′, 5′-GGGGTACCCCCTTTTTTTTTTAAAAGTCGGC-3′, 5′- GGGGTACCTGGAATTTGATATTCATTGATCCG -3′, 5′- GGGGTACCTGTATTGTTTCTC GTTTTAATTT- 3′ for the − 388, − 338, − 172 and − 104 to − 1 bp fragments respectively and the common reverse primer 5′- CCCAAGCTTAAAATCCACAGTGATTTGGGGAA - 3′. We then created a pGL4.11-VEGFpromMUT (− 388–1) mutated in two SP1 binding sites (GGGCGG ➔ GGAAGG) by amplifying the pGL4.10-VEGFprom (− 1000–1) with the forward primer 5′- GGGGTACCCCGGGAAGGGCCGGGGAAGGGG TCC -3′ (GC ➔ AA) and the reverse primer used in previous experiments. Subsequently, all the PCR products were cloned in *Kp*nI and *Hin*dIII (Amersham Biosciences) restriction sites of pGL4.11 vector. All the plasmids generated in the laboratory were sequenced by Eurofins Genomics sequencing service.

### Luciferase assay

HEK293T cells were plated at the density of 35*10^4^ cells per 35-mm-diameter culture dish and processed 40 h after the Calcium phosphate transfection. Specifically, cells were transfected with 200 ng of the specific Luciferase reporter construct, 600 ng of pEGFP-N1 HMGA1a or/and 600 ng pEGFP-FOXM1 and 10 ng of pRL-CMV Renilla expression vector (Promega), as a normalizer for transfection efficiency. The luciferase assay was also carried out by transfecting 30 pmol of HMGA1 siRNA/35-mm dish followed, 24 h later, by the transfection of 200 ng of the reporter construct and 600 ng of pEGFP-FOXM1. The Dual-Luciferase® Reporter Assay System (Promega) was used for the luciferase reporter assay, following the manufacturer instructions. The measurements were carried out using the Berthold Lumat LB 9501 Tube Luminometer; two technical replicates were performed for each sample.

### Immunoblotting

Cells were washed in ice-cold PBS and then lysed in SDS sample buffer, supplemented with proteases inhibitors, as reported before. Total lysates were separated by SDS-PAGE and then transferred to nitrocellulose membrane ∅ 0.2 μm (GE Healthcare, WhatmanTM). Western blot analyses were performed according to standard procedures, using the following antibodies: αHMGA1 [13]; α-β-actin (A2066, Sigma); αFOXM1 (A301-533A-M, Bethyl Laboratories; D3F2B Cell Signaling Technology); αGFP (kind gift of L. Collavin, LNCIB, Trieste).

### Co-immunoprecipitation

MDA-MB-231 cells protein extracts were prepared in an IP buffer containing 25 mM Tris/HCl pH 7.4, 0.5% v/v NP40 and 125 mM NaCl supplemented with 1 mM PMSF, 1 mM Na_3_VO_4_, 5 mM NaF and protease inhibitors cocktail (Sigma). For the Co-IP experiment, 250 μg of cell lysate was incubated with 1 μg of either α-HMGA1 or α-GFP (GTX113617 Genetex), as a negative control, in IP buffer O/N at 4 °C. Subsequently, pre-washed A/G proteins agarose beads (GE Healthcare) were blocked in PBS 0.1% Bovine serum albumin (BSA-Sigma) and 100 μl of beads in IP buffer were added to the solution of lysate/antibody and incubated 2 h at 4 °C. After the incubation, the beads were washed three times in IP buffer and proteins were eluted by boiling the beads for 4 min in SDS sample buffer and subjected to western blot analysis with the indicated antibodies.

### Immunostaining

HEK293T, MDA-MB-231 and MDA-MB-157 cells were grown on glass coverslips and silenced for HMGA1 and/or FOXM1, as previously described. HEK293T cells were transfected with 1 μg of pEGFP-FOXM1 1 day post HMGA1-silencing, by Calcium phosphate method. Then cells were fixed in a solution of 4% PFA. After a permeabilization with 0.3% Triton/PBS and saturation in 5% BSA/PBS, cells were incubated with the following primary antibodies: αFOXM1 (Cell Signaling Technology); α-20S Proteasome β2 (MCP165 - sc-58410); α-20S Proteasome β5 (A10 - sc-393931). The α-rabbit and α-mouse Alexa 594 and 488 were used respectively. The images were visualized using a Nikon Eclipse e800 microscope and acquired using Nikon ACT-1 software, then analyzed by the ImageJ software analyser.

### Migration assay

For the wound healing assay, MDA-MB-231 and MDA-MB-157 cells were seeded in antibiotics-free DMEM at a density of 2*10^5^ cells/well in a 35-mm dish in biological triplicates and silenced for HMGA1 and/or FOXM1 expression, as described before. Cells were cultured to 90% confluence and then scraped with a 200-μl tip, and wound closure has been followed for 7 h. Two images for the same area were taken for each well and the wound areas were analyzed by ImageJ software.

### Gene expression analysis

Total RNA from MDA-MB-231 cells was isolated following the manufacturer’s instructions of the TRIzol reagent (Thermo Fisher Scientific), subjected to DNase-I (Thermo Fisher Scientific) treatment and subsequently purified using phenol-chloroform. For quantitative RT-PCR (qRT-PCR), mRNA was reverse transcribed with Random primer by the Superscript III (Thermo Fisher Scientific), according to the manufacturer’s instructions. qRT-PCR was carried out with iQ™ SYBR Green Supermix (BIO-RAD); specific primers used are listed in Additional file 1: Table S1. The data obtained were analyzed with BIO-RAD CFX Manager software and the relative gene expression was calculated by the ΔΔCt method, using the GAPDH as a normalizer.

### Preparation of cells for RNA-sequencing analysis

MDA-MB-231 cells were silenced for HMGA1 and the RNA was collected at 24 and 72 h after the silencing. Three biological replicates were made for each condition. The total RNA was then extracted and checked as described above. Then, an aliquot of RNA was reverse transcribed and the silencing of HMGA1 was confirmed by qRT-PCR.

### RNA-sequencing analysis

Demultiplexed raw reads (fastq) generated from the Illumina HiSeq were checked using FASTQC tool (Version 0.11.3). All samples passed the quality standards. Then we aligned them to the reference genome (UCSC-hg19) using STAR [25], version 2.0.1a using recommended options and thresholds. HTSeq-count (version 0.6.1) was used to generate gene counts. Starting from read counts, differential gene expression analysis was performed using EdgeR (version 1.10.1, R version: 3.2.3, [26]) comparing the different time points using a quantile-adjusted conditional maximum likelihood (qCML) method. In order to identify the relationship between each sample and every other sample, the Euclidean distance between each pair of samples was calculated using the log-transformed values of the complete dataset. Average linkage clustering was then used to generate a sample-to-sample distance heatmap, via the cluster3 package (Cluster3: http://bonsai.hgc.jp/~mdehoon/software/cluster/software.htm#ctv) [27]. For statistical analyses the adjusted *p*-values were generated via the Benjamini-Hochberg procedure. Finally, genes were selected as differentials with a cutoff of 0.5 for the log Fold change and 0.05 for the False Discovery Rate.

### Functional analysis of differentially expressed genes

Differentially expressed genes were analyzed using GSEA [28, 29] and Ingenuity Pathway Analysis (IPA, Ingenuity® Systems, www.ingenuity.com) [30]. The prediction of the transcription factors was obtained using the “upstream regulators” module (IPA suite). For every upstream regulator an overlap *p*-value and a z-score were calculated: the *p*-value indicates the significance based on the overlap between dataset genes and known targets regulated by the molecule, while the z-score is used to infer the possible activation (z-score ≥ 1.8) or inhibition (z-score ≤ − 1.8) of the molecule based on prior knowledge stored in the proprietary Ingenuity Knowledge Base. All statistical test and calculation have been performed in R [31] environment. For the enrichment in protein localization, genes were annotated using Uniprot (https://www.uniprot.org) and proteins were analyzed with David/Ease [32, 33] interrogating the Geoterm Cellular Compartment gene ontology. For Fig. 6a) we selected the most significant terms in enrichment clusters (enrichment score > 3) including nuclear, microtubules, adherence/junction and secreted/exosome terms.

### Preparation of the conditioned medium (CM) for angiogenic assays

MDA-MB-231 were seeded and silenced for HMGA1 and/or FOXM1 as described and 1 day before the supernatant collection the culture medium was substituted with serum-free DMEM. After 72 h of silencing, the CM was collected, centrifuged at 240Xg at 4 °C for 5 min to deposit cells debris and stored at − 80 °C. Next, the cells were washed in PBS 1X and lysed in SDS sample buffer as described. The HMGA1 and FOXM1 protein levels were checked by western blot analysis.

### Endothelial cells proliferation analysis

Human umbilical vein endothelial cells (HUVEC) have been isolated from human umbilical cords [34] and seeded in 96-well plate at the density of 5*10^3^ cells/well. The cells were incubated with HMGA1, FOXM1 and HMGA1/FOXM1 conditioned medium and the control medium for 18 h. Normal Human Serum 10% was used as conditioned medium for the positive control experiments. After a washing step, the cells were fixed and permeabilized with Fix&Perm kit (Nordic-MUbio) for 15 min at room temperature in the dark. After two washing steps, the cells were incubated with the α-Ki-67 antibody (Dako), followed by the incubation with the secondary antibody α-mouse FITC (Dako). The cells were washed twice and lysed. Measurements were performed using Infinite200 (Tecan).

### Transwell migration assay of endothelial cells

HUVEC cells were seeded in 200 μl of Endothelial serum-free medium (Invitrogen) at the density of 15*10^4^ cells /well in the upper compartment of 8 μm pore 24-transwell plate, pre-coated with human Fibronectin (Sacco) in the lower face. Then HUVEC were incubated with 500 μl of HMGA1 and/or FOXM1 CMs, and with serum-free medium as a negative control or Normal Human Serum 10% (NHS) as a positive control in the lower compartment of the transwell. After 18 h of incubation, HUVEC were lysed with lysis buffer for Coulter and the number of migrated cells were counted with Coulter Counter BD.

### In vitro tube formation of endothelial cells

HUVEC at the density of 5.5*10^4^ were plated on wells precoated with Matrigel (12 μg/ml) (Becton Dickinson) and incubated for 18 h with CMs diluted 1:2, with 20 ng/ml VEGF as a positive control or with serum-free medium as a negative control. After a 4% paraformaldehyde fixation step and staining with Phalloidin-Alexa Fluor 546 (Invitrogen), the number of tubules was counted under a Leica AF6500 microscope using LAS software (Leica).

### Preparation of cells for zebrafish injection

MDA-MB-231 cells were seeded at the density of 1.2*10^6^ per 10-mm-diameter culture dish and silenced for HMGA1 and/or FOXM1 as described before. After 40 h of silencing the cells were counted and injected in the yolk sack of zebrafish embryos.

### Zebrafish xenograft

Zebrafish were raised and maintained as previously described [35]. Embryos were generated by natural pair-wise mating and were kept and handled for all experiments in E3 medium (5 mM NaCl, 0.17 mM KCl, 0.33 mM CaCl_2_, 0.33 nM MgSO_4_) and PTU (1-phenyl 2-thiourea, 0.03 mg/ml) to reduce zebrafish skin pigmentation for microscope analysis. All experimental procedures were performed conforming to the ITA guidelines (Dgl 26/2014) in accordance with EU legislation (2010/63/UE); this protocol was approved by a committee of the Italian Health Ministry (cod. 04086.N.15Y). In this study, the Tg (fli1:EGFP) (y1) zebrafish embryos were used. This transgenic fluorescent strain expresses in the entire vasculature EGFP under the control of the fli1 promoter [36]. Before all experimental procedures, animals were properly anesthetized by using 1:100 dilution of 4 mg/ml Tricaine (Sigma-Aldrich Co., St Louis, MO, USA). Two days post fertilization (2dpf), zebrafish embryos were dechorionated and microinjected with MDA-MB-231 siCTRL and siHMGA1/siFOXM1 cells or cellular medium alone (vehicle) as control experiment. Each cells suspension was stained by 2 μg/ml DiI (Sigma Aldrich) for 10 min at 37 °C, re-suspended in DMEM medium and kept on ice before injection. Microinjections were performed with the electronic FemtoJet microinjector (Eppendorf) using borosilicate glass micro-capillaries (20 mm O.D. Fifteen millimeters I.D.; Eppendorf). Approximately 500 cells were microinjected into the yolk of each embryo, which was then maintained in E3 medium/PTU for 1 h at 28 °C. Afterwards embryos were kept at 34 °C to allow tumor cells survival and growth as previously described [37]. Twenty-four hours after microinjection, the embryos were observed with a fluorescence microscope (Leica DM 2000). During all the procedures living animals, properly anesthetized, were positioned in 1.5% methylcellulose (Sigma Aldrich Co). The images of the tumor masses were acquired in red signal (DiI staining) and then merged with the respective bright field image by using the Leica Application Suite X (LAS X) software. Instead, to evaluate the host angiogenic response to the injected tumor cells, vessels images were acquired in green (GFP) signal and then analyzed using the ImageJ software. For both analyses, 30 animals for each cell line condition, divided into two independent experiments, were considered. For gene expression, total RNA was extracted from at least 20 embryos for each experiment using Trizol Reagent (Invitrogen, Life Technologies, Milan, Italy). RNA concentration was determined by Nanoquant (Tecan). One microgram of total RNA was reverse transcribed using M-MLV reverse transcriptase (Life Technologies). Gene expression was analyzed by qRT-PCR (Step One Plus, Applied Biosystems) using the SYBR Green system (Life Technologies). The primer sequences are listed in Additional file 1: Table S1. The gene analysis was repeated three times and the values were normalized respect to medium treated animals.

### Breast cancer dataset

Data from breast cancer patients have been obtained from the TCGA BRCA dataset (updated to September 2018) using the cgdsr package for R. Survival analysis has been performed using Kaplan Meier plotter [38] with distant metastasis-free survival (DMFS) as a read-out on a cohort of 1746 breast cancer patients and relapse-free survival (RFS) as a read-out on cohorts of 3951 breast cancer patients or 255 TNBC patients.

### Statistical analysis

Data were analyzed by a two-tailed Student’s t-test, and results were considered significant at a *p*-value < 0.05. The results are presented as the mean and standard deviation (±SD). Specifically, a *p*-value< 0.05 is indicated with *, a *p*-value< 0.01 with ** and a *p*-value< 0.001 with ***.

## Results

### Identification of upstream regulators of HMGA1 breast cancer gene networks

**Fig. 1:**
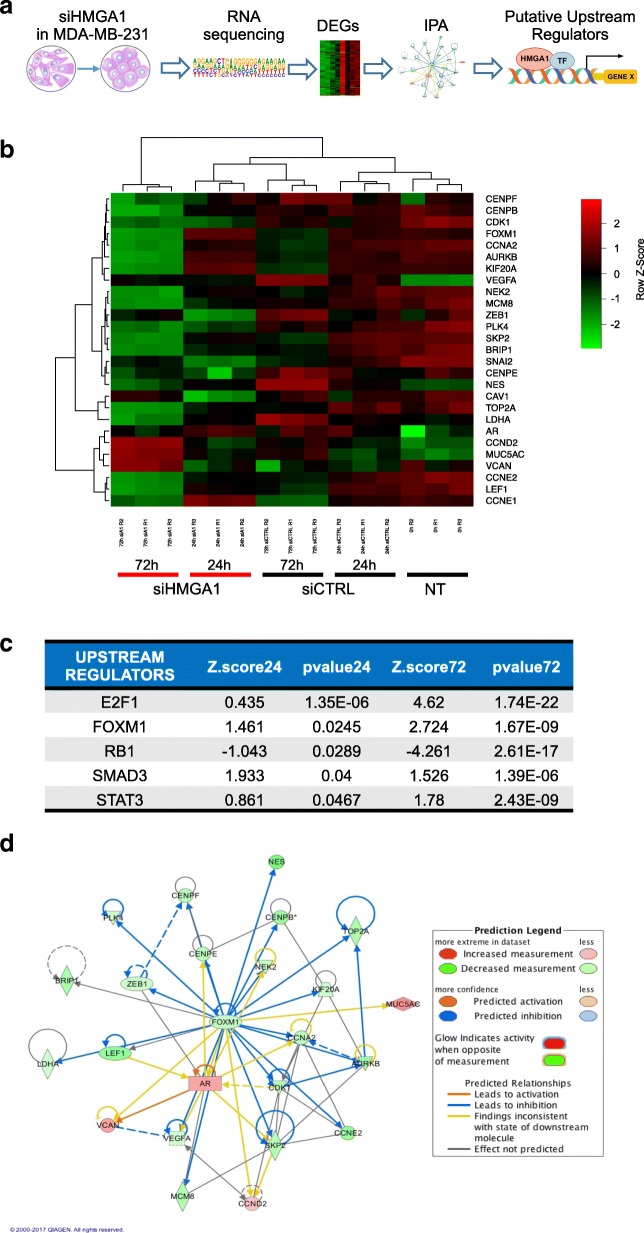
Identification of upstream regulators of HMGA1 breast cancer gene networks. **a** The flow-chart shows the main steps used to identify new molecular partners (TF in figure) of HMGA1, in regulating cancer-related gene networks, starting from RNA-Seq data. **b** Heatmap showing the expression of the top differentially expressed genes (DEGs) upon HMGA1 silencing after 24 and 72 h in MDA-MB-231 cells. Color intensity corresponds to the row Z-Score. **c** Pathways associated to DEGs were rebuilt by Ingenuity Software analysis. Using the Upstream regulators function (IPA suite) the transcription factors and regulative molecules of these networks were predicted. The figure shows the list of the top 5 transcription factors, putative molecular partners of HMGA1, ranked by their 24 h *p*-value (obtained from 24 h data). **d** Visual representation of Ingenuity pathway interactive-network of FOXM1. The connected-factors are found activated or inhibited by HMGA1 from RNA-Seq analysis

We previously demonstrated the pivotal role of HMGA1 in conferring several cancer hallmarks in TNBC, showing that HMGA1 is involved in modulating an intricate molecular network [13]. To better define HMGA1-dependent molecular networks in sustaining breast cancer aggressiveness, we analyzed global gene expression profiles after HMGA1 depletion in the MDA-MB-231 TNBC cell line. To this end, we silenced the expression of HMGA1 by transfecting cells with siRNAs specific for HMGA1 (siHMGA1) or with no-target control siRNAs (siCTRL) as previously described [13]. Then, RNA was isolated at two time points after transfection (24 and 72 h) and the RNA was subjected to RNA-Seq analysis. To identify HMGA1-molecular partners potentially critical in governing breast cancer aggressiveness we proceeded with a bioinformatic analysis that consisted mainly of three steps: 1. identification of differentially expressed genes (DEGs) upon HMGA1 silencing; 2. analysis of gene networks in which DEGs are involved; 3. prediction of transcription factors regulating these gene networks (Fig. 1a).

After the identification of the transcripts whose expression changed upon HMGA1 depletion at the different time points (Fig. 1b and Additional file 2: Figure S1), we analyzed the potential pathways regulated by HMGA1 by interrogating the Ingenuity Pathway software (IPA). This tool crosses the gene expression changes observed with known datasets collected from literature data to build up the gene networks in which these DEGs are included. Next, using the “upstream regulators” module of IPA, we predicted the regulative molecules that could govern the gene networks modulated by HMGA1. To each upstream factor identified, an overlap *p*-value (a measure of how much the overlap between their known dataset and our HMGA1-modulated gene list is significant) and a z-score (an indicator of the state of activation/inhibition of the gene network when the molecule we identified as hub is active) were assigned. We considered as upstream regulators of the HMGA1-molecular network the factors with a z-score ≤ − 1.8 or ≥ 1.8 in at least one time point analyzed. Then, we decided to focus on transcription factors as putative molecular partners of HMGA1 (Fig. 1c). Interestingly, E2F, RB1 and STAT3 have already been identified as HMGA1-molecular partners [39, 40] confirming the reliability of our approach. Specifically, we found that when HMGA1 is active the molecular network of RB1 is inhibited (negative z-score) whereas that of E2F is activated (positive z-score) (Fig. 1c). This is consistent with the notion that HMGA1 binds RB1, inducing in this way the release of E2F and the consequent activation of E2F-dependent transcription [40]. This observation is also supported by GSEA analysis from both the 24 and 72 h HMGA1 silencing data (Additional file 3: Tables S2 and S3). For further analysis, we focused on FOXM1, a transcription factor involved in cell cycle regulation with a pivotal role in cancer initiation and progression [41].

IPA analysis showed that FOXM1 is a hub of HMGA1-dependent gene networks (Fig. 1d), thus revealing that several genes found to be differentially expressed at 24 and 72 h after HMGA1 silencing were also predicted to be downstream targets of FOXM1 (Additional file 4: Table S4). These data suggest that HMGA1 could cooperate with FOXM1 in regulating specific gene networks fundamental in sustaining breast cancer aggressiveness.

### FOXM1 is a new molecular partner of HMGA1 in breast cancer progression

**Fig. 2:**
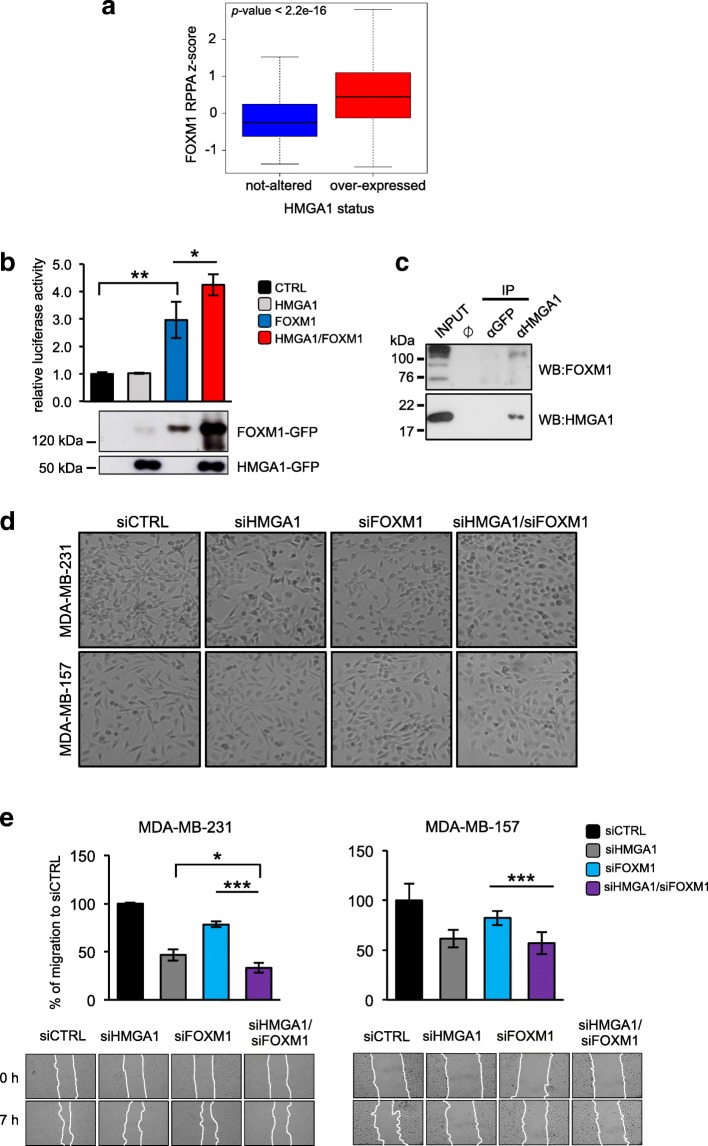
FOXM1 is a new molecular partner of HMGA1 in breast cancer progression. **a** Boxplots showing the FOXM1 protein levels in breast cancer patients. The samples were stratified based on HMGA1 mRNA expression levels. **b** HEK293T cells were transiently co-transfected with the luciferase reporter plasmid pGL3-5BS (see Additional file 5: Figure S2b for promoter reporter diagram), the expression plasmid pEGFP-HMGA1 (grey bar), pEGFP-FOXM1 (blue bar) or the combination of two (red bar). pRL-CMV Renilla luciferase expression vector was included to normalize for transfection efficiencies. Values are reported as relative luciferase activity compared to cells transfected with the expression vector pEGFP (black bar). The data are represented as the mean ± SD (*n* = 3); *p < 0.05, **p < 0.01; two-tailed Student’s *t*-test. Below the graph, representative western blot validations of HMGA1-GFP and FOXM1-GFP over-expressions are shown. **c** Co-immunoprecipitation (co-IP) of FOXM1 and HMGA1. The experiment was performed with either the negative control α-GFP or the α-HMGA1 antibodies on MDA-MB-231 cells lysates. Inputs (15% of the amount used for IP) and the immunoprecipitated proteins were subjected to western blot analysis with the α-HMGA1 and the α-FOXM1 (Bethyl Laboratories) antibodies. **d** Representative pictures of cell morphology of MDA-MB-231 (upper figures) and MDA-MB-157 (lower figures) in control condition and after HMGA1, FOXM1 and HMGA1/FOXM1 depletion (siHMGA1, siFOXM1 and siHMGA1/siFOXM1). **e** Wound healing assay of MDA-MB-231 and MDA-MB-157 silenced for HMGA1 and/or FOXM1. The wound areas were measured with ImageJ software and results are plotted on the graphs. The data are represented as the mean of percentage of the wound closure relative to siCTRL ±SD (*n* ≥ 3), *p < 0.05, ***p < 0.001; two-tailed Student’s *t*-test. Below the graphs, representative images of wound closure, taken at 4X magnification, are reported

Given the potential role of FOXM1 as a molecular partner of HMGA1, we searched for a relationship between HMGA1 and FOXM1 in The Cancer Genome Atlas (TCGA), a public database collecting information from a high number of cancer patients obtained from high-throughput approaches. From this catalogue, we selected a dataset of 818 breast cancer patients, and we found that FOXM1 was enriched in HMGA1 overexpressing patients, both at mRNA and protein levels (Additional file 5: Figure S2a and Fig. 2a). To explore a possible cooperation between HMGA1 and FOXM1, we investigated the effects of HMGA1 on FOXM1 transcriptional activity in HEK293T cells using a FOXM1-responsive luciferase reporter with 5 binding elements for FOXM1 (Additional file 5: Figure S2b). HMGA1 itself was not able to activate the reporter gene but it increased FOXM1-transcriptional activity (Fig. 2b), indicating a relationship between these two factors in the transcriptional regulation. Notably, we observed an increase of exogenous FOXM1 protein following HMGA1 transfection, suggesting that HMGA1 could regulate FOXM1 at a post-transcriptional level (Fig. 2b). This effect is tightly dependent on HMGA1 presence, indeed, transfecting HEK293T cells with a fixed quantity of FOXM1 and increasing amounts of HMGA1, we detected increased levels of exogenous FOXM1 protein in a HMGA1 dose-dependent manner (Additional file 5: Figure S2c). Given these results we checked whether HMGA1 physically interacts with FOXM1. To this end, we performed co-immunoprecipitation (Co-IP) of endogenous proteins in MDA-MB-231 cells, revealing the presence of FOXM1 in the HMGA1 immunocomplex (Fig. 2c).

To better elucidate the cooperative action of HMGA1 and FOXM1 in breast cancer cells, we performed qRT-PCR on RNA derived from the TNBC MDA-MB-231 cell line in which we silenced the expression of HMGA1 or FOXM1 or the two factors in combination (Additional file 5: Figure S2d) and we analyzed the expression of CCNE2, LEF1 and VEGFA that emerged, from our bioinformatics analysis, as common target genes of HMGA1 and FOXM1 (Additional file 4: Table S4). We found that the expression of all tested genes is downregulated after both HMGA1 and FOXM1 silencing and that, interestingly, their expression is even more downregulated when both factors were silenced (Additional file 5: Figure S2e). These results confirmed that HMGA1 and FOXM1 are molecular partners and that they act cooperatively in the regulation of gene transcription.

Since the target genes analyzed are key factors of several cancer hallmarks such as EMT, migration and angiogenesis, we next considered the possibility that HMGA1 and FOXM1 could act in concert in mediating cancer-related cell abilities. To this end, we took into consideration the same conditions analyzed for qRT-PCR and we observed that the concomitant depletion of HMGA1 and FOXM1 brought the cells to a more epithelial phenotype than single silenced conditions in two TNBC cell lines, MDA-MB-231 and MDA-MB-157 (Fig. 2d). Moreover, we performed scratch migration assay and we found that the concomitant depletion of HMGA1 and FOXM1 decreased migration properties of MDA-MB-231 and MDA-MB-157 with more efficiency than the single silenced conditions (Fig. 2e and Additional file 5: Figure S2f). Thus, our results suggest that HMGA1, by interacting with FOXM1, could stabilize FOXM1 protein and increase its transcriptional activity; this cooperative action is fundamental in conferring aggressive features in TNBC cells.

### HMGA1 regulates FOXM1 at a post-transcriptional level via the proteasome pathway

**Fig. 3:**
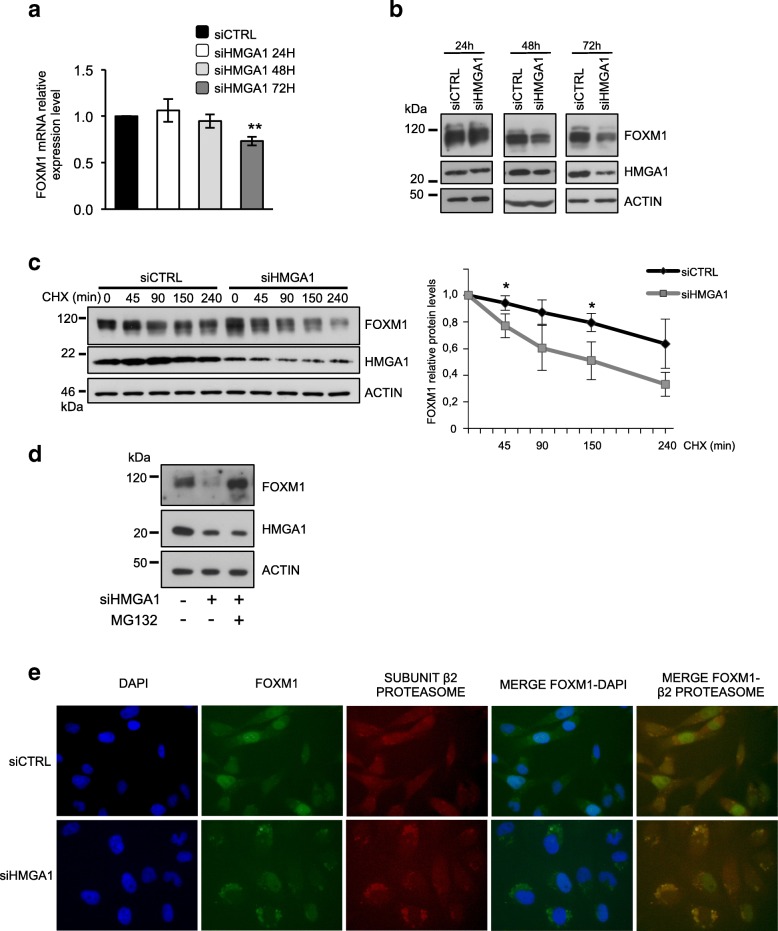
HMGA1 regulates FOXM1 at a post-transcriptional level via the proteasome pathway. **a** qRT-PCR of FOXM1 mRNA level in MDA-MB-231 cells after 24, 48 and 72 h after HMGA1 silencing. GAPDH was used for normalization. The data are compared to the control condition and are presented as the mean ± SD (*n* ≥ 3), **p < 0.01; two-tailed Student’s *t*-test. **b** Western blot analysis of FOXM1 protein level in MDA-MB-231 cells upon 24, 48 and 72 h of HMGA1 silencing. β-actin was used as a loading control. **c** Western blot analysis of FOXM1 protein levels in MDA-MB-231 control cells and cells silenced for HMGA1, treated with cycloheximide (CHX) for the indicated times. The FOXM1 band intensity was normalized to total protein lysate stained with Red Ponceau, based on ImageJ quantification. The protein levels of FOXM1 relative to the time point 0 of each group were calculated and are shown in the graph on the right, *n* = 3, *p < 0.05; two-tailed Student’s *t*-test. **d** Western blot analysis of FOXM1 protein level in MDA-MB-231 cells silenced for HMGA1 and treated with the proteasome inhibitor MG-132. **e** Representative immunofluorescence images of the translocation of FOXM1 (green) and its colocalization with the Subunit β2 of the proteasome (red) in MDA-MB-231 control cells versus cells silenced for HMGA1. Images were taken at 60X magnification

Different reports describe that FOXM1 is regulated at multiple levels and that its regulation is crucial for its activity and cancer development [42]. Therefore, we decided to go deeper in investigating the mechanisms by which HMGA1 regulates FOXM1. Thus, we silenced the expression of HMGA1 in MDA-MB-231 cells and we evaluated the expression of FOXM1 at mRNA and protein levels at different time points. At 24 and 48 h after HMGA1 silencing, the FOXM1 mRNA level remained unchanged and only at 72 h it was slightly downregulated (Fig. 3a). Instead, at the protein level, already at 48 h we observed a reduction in FOXM1 expression that became stronger at 72 h (Fig. 3b), confirming the above-described observation on the regulation of FOXM1 at the post-transcriptional level.

Taking into account that one of the levels of FOXM1 protein regulation is its nucleo-cytoplasmic shuttling and subsequent degradation [43], we investigated FOXM1 localization after HMGA1 silencing in MDA-MB-231 and MDA-MB-157 cells. We observed that the silencing of HMGA1 caused a more cytoplasmic localization of FOXM1 with respect to control condition in which FOXM1 is predominantly nuclear (see FOXM1 staining in Fig. 3e and Additional file 6: Figure S3a-c). We confirmed these results by exploring the localization of transfected FOXM1-GFP in MDA-MB-231 and in HEK293T cells upon HMGA1 silencing: indeed, after HMGA1 depletion, FOXM1-GFP shows a cytoplasmic localization (Additional file 6: Figure S3d and e). These results suggest that HMGA1 could modulate the stability of FOXM1 protein by controlling its localization.

Then, we compared FOXM1 protein levels between HMGA1-silenced and control MDA-MB-231 cells after cycloheximide (CHX) treatment. We observed that, in the presence of CHX, the half-life of FOXM1 protein was shorter in HMGA1-silenced cells with respect to control cells (Fig. 3c), showing a lower stability of FOXM1 in the absence of HMGA1. Next, we asked whether HMGA1 might modulate FOXM1 protein degradation and therefore we exposed cells to the 26S proteasomal inhibitor MG132. The treatment of MG132 rescued the FOXM1 levels that were reduced by HMGA1-silencing (Fig. 3d). Consistently, we observed that, in MDA-MB-231 and -157 cell lines silenced for HMGA1, FOXM1 co-localized with the proteasome subunits β2 and β5 (Fig. 3e and Additional file 6: Figure S3 a-c). All together our evidence indicates that HMGA1 prevents FOXM1 proteasomal degradation and stabilizes it in the nucleus.

### HMGA1 increases the transcriptional activity of FOXM1 on VEGFA promoter

Among the genes belonging to the HMGA1/FOXM1-gene network, we found VEGFA. Vascular Endothelial Growth Factor is a potent promoter of angiogenesis- the formation of a novel vasculature system from a pre-existing one- both in physiological and pathological conditions such as cancer. Given its crucial role, it is not surprising that VEGFA expression is finely regulated at different levels including transcription. In fact, multiple response elements, several trans-activating factors and many stimuli are known to regulate VEGFA transcription [44]. Some reports underline the involvement of HMGA1 and FOXM1 in controlling VEGFA expression in different biological contexts [45, 46]. Moreover, in a TCGA dataset of breast cancer patients, we found an enrichment of VEGFA mRNA in HMGA1 or FOXM1 overexpressing patients (Additional file 7: Figure S4a).

**Fig. 4:**
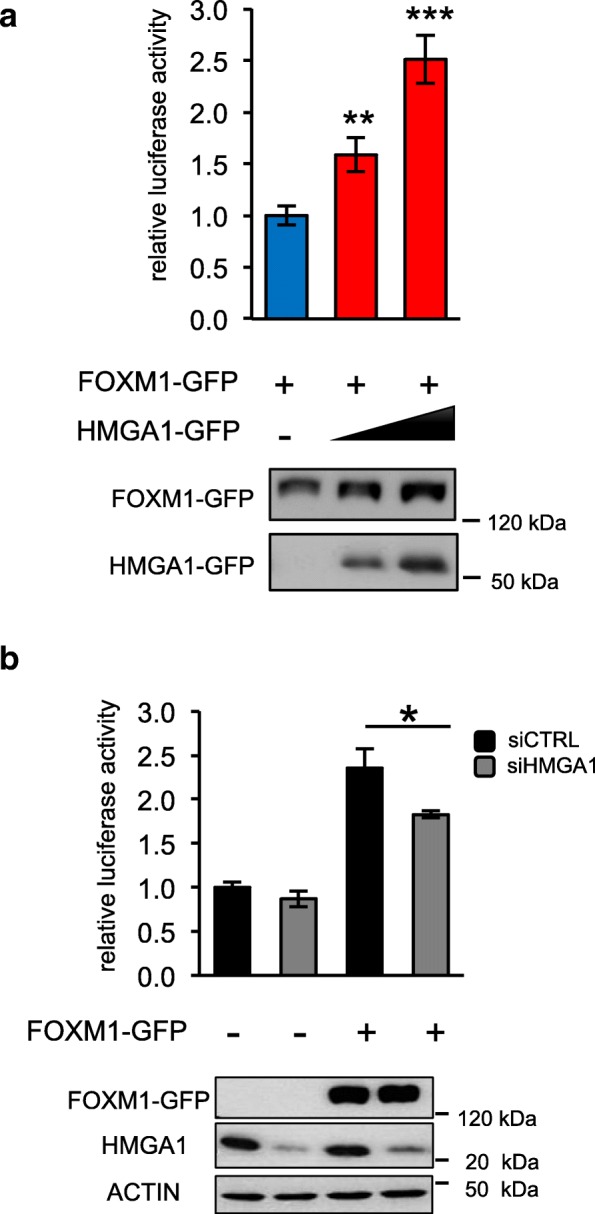
HMGA1 increases the transcriptional activity of FOXM1 on VEGFA promoter. **a** Luciferase assay on HEK293T cells transiently co-transfected with the luciferase reporter plasmid pGL4.10-VEGFprom (− 1000–1) and with pEGFP-FOXM1 and increasing quantities of pEGFP-HMGA1 expression vectors (red bars). pRL-CMV Renilla luciferase expression vector was included to normalize for transfection efficiencies. Values are reported as relative luciferase activity compared to cells transfected with pEGFP-FOXM1 in the absence of pEGFP-HMGA1 and are presented as the mean ± SD (*n =* 3). **p < 0.01, ***p < 0.001; two-tailed Student’s *t*-test. Below the graph, the correspondent western blot validations of HMGA1 and FOXM1 protein levels are reported. **b** Luciferase assay on HEK293T cells silenced for HMGA1 by siRNA and transiently co-transfected with the luciferase reporter plasmid pGL4.10-VEGFprom (− 1000–1) with the expression plasmid pEGFP-FOXM1. pRL-CMV Renilla luciferase expression vector was included to normalize for transfection efficiencies. Values are reported as relative luciferase activity compared to cells transfected with the control expression vector pEGFP. The data are represented as the mean ± SD (*n =* 3), *p < 0.05; two-tailed Student’s *t*-test. Below the graph, the correspondent western blot validation. β-actin was used as a loading control for endogenous HMGA1

To deepen the mechanism by which HMGA1 and FOXM1 regulate VEGFA expression, we evaluated their transcriptional activity on a luciferase reporter vector containing a portion of the VEGFA promoter spanning from − 1000 to − 1 bp from the Transcriptional Start Site (TSS) [47]. We transfected this vector along with expression vectors for HMGA1 or FOXM1 in HEK293T cells. In agreement with literature data, both HMGA1 and FOXM1 increased VEGFA-luciferase activity in a dose-dependent manner (Additional file 7: Figure S4b and c). To evaluate the contribution of HMGA1 on FOXM1 transcriptional activity, we co-transfected FOXM1 and increasing amount of HMGA1 expression vectors and we showed that HMGA1 enhanced FOXM1 transcriptional activity on VEGFA promoter in a dose-dependent manner (Fig. 4a). Consistently, by silencing the expression of endogenous HMGA1 and transfecting FOXM1 expressing vector, we observed that HMGA1 silencing diminished the transcriptional activity of FOXM1 on VEGFA promoter, confirming the role of HMGA1 on FOXM1-transcriptional activity (Fig. 4b).

### HMGA1 regulates VEGFA promoter with two independent ways: via Sp1 and FOXM1

**Fig. 5:**
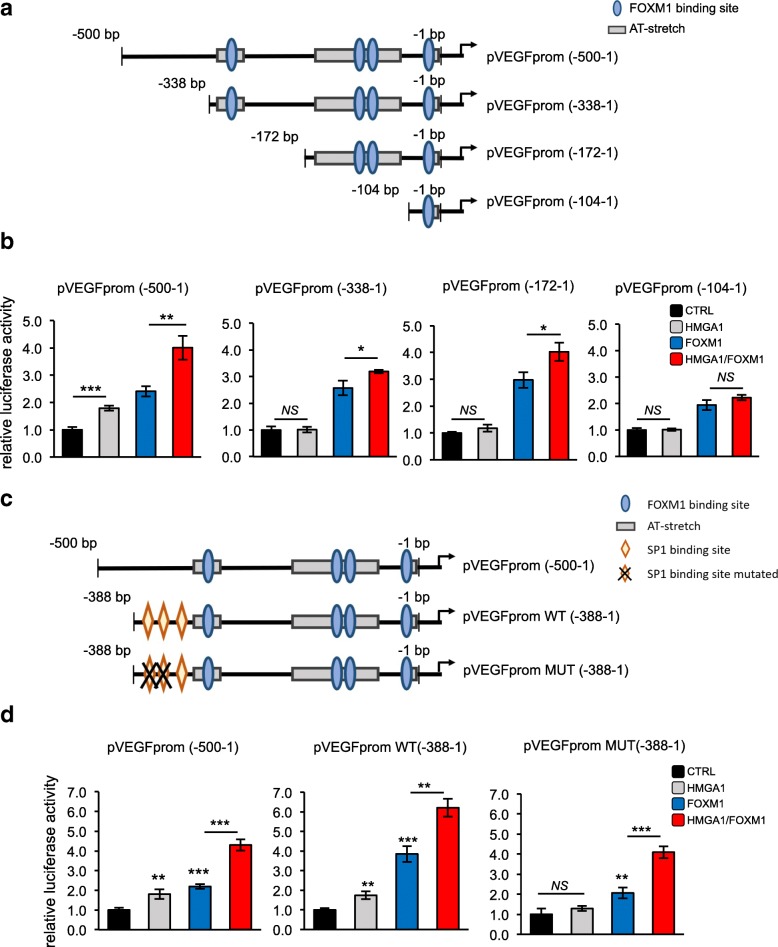
HMGA1 regulates the VEGFA promoter with two independent ways: via Sp1 and FOXM1. **a** Schematic representation of the VEGFA deletion mutant reporter vectors obtained with progressive deletions of the pGL4.10-VEGFprom (− 500–1) and used in the experiments. FOXM1 binding sites are represented with light blue ovals, whereas the AT-enriched sequences bound by HMGA1 are shown as grey boxes. **b** Luciferase assay on HEK293T cells transiently co-transfected with the luciferase reporter plasmid pGL4.10-VEGFprom (− 500–1), the deletion mutant vectors pGL4.11-VEGFprom (− 338–1), pGL4.11-VEGFprom (− 172–1) or pGL4.11-VEGFprom (− 104–1) with the expression plasmid pEGFP-HMGA1 (grey bar), pEGFP-FOXM1 (blue bar) and pEGFP-HMGA1/pEGFP-FOXM1 (red bar). pRL-CMV Renilla luciferase expression vector was included to normalize for transfection efficiencies. Values are reported as relative luciferase activity compared to cells transfected with the correspondent reporter vectors and the expression plasmid pEGFP. The data are represented as the mean ± SD (*n* = 3). *p < 0.05, **p < 0.01, ***p < 0.001, *NS*: Not Significant; two-tailed Student’s t-test. An example of western blot validations is reported in Additional file 8: Figure S5d. **c** Schematic representation of pGL4.10-VEGFprom (− 500–1) and deletion reporter vectors, pGL4.11-VEGFprom (− 388–1) wild-type (WT) and mutated in SP1-binding sites (MUT). FOXM1 binding site is represented with light blue oval, the AT-enriched sequence bound by HMGA1 is figured as grey box, the orange rhombus represents the SP1 binding element and the black cross indicates the mutation of SP1 binding element. **d** Luciferase assay on HEK293T cells transiently co-transfected with the luciferase reporter plasmids pGL4.10-VEGFprom (− 500–1), pGL4.11-VEGFpromWT (− 388–1) or pGL4.11-VEGFpromMUT(− 388–1) with the expression plasmids pEGFP-HMGA1(grey bar), pEGFP-FOXM1 (blue bar) or pEGFP-HMGA1/ pEGFP-FOXM1 (red bar). pRL-CMV Renilla luciferase expression vector was included to normalize for transfection efficiencies. Values are reported as relative luciferase activity compared to cells transfected with the reporter vectors used and the expression plasmid pEGFP. The data are represented as the mean ± SD (*n* = 3). **p < 0.01, ***p < 0.001, two-tailed Student’s t-test. An example of western blot validations is reported in Additional file 8: Figure S5e

Subsequently, we asked where HMGA1 and FOXM1 exert their transcriptional action on the VEGFA promoter. To this end, we analyzed the − 1000 to − 1 bp VEGFA promoter region by searching potential binding sites for the two factors. Specifically, we looked for AT-rich regions with at least three consecutive A/T as a putative binding site for HMGA1 [48], and the Forkhead proteins family common binding sequence RYAAAYA (R = A or G and Y=C or T) for FOXM1 [49]. We found several AT-rich sequences and 9 putative FOXM1 binding sites (Additional file 8: Figure S5a). On the bases of the promoter analysis carried out, we explored whether the cooperative action between HMGA1 and FOXM1 occurs in the VEGFA promoter region spanning from − 1000 to − 500 or the subsequent region from − 500 to − 1 bp from the TSS (Additional file 8: Figure S5b). We co-transfected the full-length promoter (− 1000–1 bp) or the two deletion reporter constructs (− 1000–500 bp and − 500-1 bp) [47] with expression vectors for HMGA1 and FOXM1. We observed that the (− 500–1 bp) construct maintained a transactivation of the reporter at high levels, comparable to the full-length promoter condition, whereas this effect was lost in the (− 1000–500 bp) VEGFA reporter vector (Additional file 8: Figure S5c). These results clearly indicate that the region spanning from − 500 to − 1 bp of VEGFA promoter is responsible for HMGA1 and FOXM1 activity. Next, on the bases of the AT-rich regions and the FOXM1 putative binding elements found with the promoter analysis, we generated three mutant reporter vectors, introducing progressive deletions in the − 500-1 bp VEGFA promoter (− 338–1 bp, − 172-1 bp and − 104-1 bp) (Fig. 5a). We transfected the above described VEGFA deletion mutant reporters together with expression vectors for HMGA1, FOXM1 and a combination of both and we evaluated their luciferase activity in comparison with the (− 500–1 bp) reporter vector. Firstly, we observed that HMGA1 was able to transactivate the (− 500–1 bp) reporter construct whereas it failed to transactivate the other three deletion constructs tested, indicating that HMGA1 alone acts preferentially in the region of the VEGFA promoter spanning from − 500 to − 338 (Fig. 5b). In addition, we observed that HMGA1 maintained its ability to potentiate the activity of FOXM1 in all the reporters except for the (− 104–1 bp) construct (Fig. 5b and Additional file 8: Figure S5d). These results suggest that HMGA1 regulates VEGFA expression in two different ways: a FOXM1-independent mechanism in the region from − 500 to − 338 bp of the VEGFA promoter and a FOXM1-dependent way, in the region from − 338 to − 104 bp, where a functional FOXM1 binding element has been previously identified [46].

Interestingly, Sp1, a well-known transcription factor, can bind VEGFA promoter in the region from − 385 to − 352 bp. Specifically, in this region three Sp1 consensus elements have been reported, the first and the second binding sites being more relevant [50]. Intriguingly, it has been described that HMGA1 interacts with Sp1 potentiating its activity in the activation of the Insulin receptor promoter [51]. Therefore, to explore whether HMGA1 cooperates with Sp1 on the VEGFA promoter, we used two deletion reporter constructs containing a region of the VEGFA promoter spanning from − 388 to − 1 bp from the TSS, and, in one of them, we mutated the two Sp1 binding sites previously described [50] (Fig. 5c). We transfected these reporters along with the (− 500–1 bp) reporter and we evaluated the transcriptional activity of HMGA1, FOXM1 and a combination of both. The results showed that while HMGA1 is able to activate the (− 338–1 bp) promoter construct at the same level of the (− 500–1 bp) one, the activity of HMGA1 on this promoter region is disrupted by the mutation of Sp1 binding sites, indicating the tight dependence of HMGA1 activity from Sp1 on this promoter region (Fig. 5d and Additional file 8: Figure S5e). Interestingly, HMGA1 is still able to enhance FOXM1 transcriptional activity on the VEGFA promoter regardless of the mutation of Sp1 binding elements (Fig. 5d). All these results together indicate that HMGA1 controls VEGFA transcription by cooperating with FOXM1 and Sp1 transcription factors and that these two ways are independent.

### HMGA1 and FOXM1 cooperative action in breast cancer cells governs angiogenic processes of endothelial cells

**Fig. 6:**
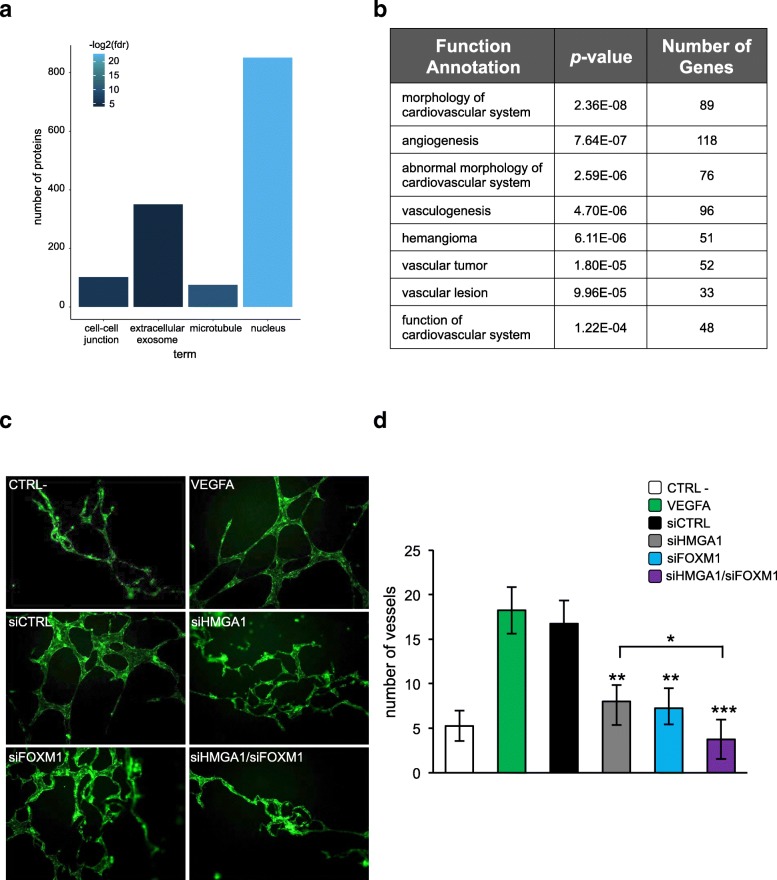
HMGA1 and FOXM1 cooperative action in breast cancer cells governs angiogenic processes of endothelial cells. **a** Functional enrichment for Cellular Compartment ontology. Color intensity corresponds to the -log2(*p*-value) of the enrichment significance. **b** A Gene ontology analysis was carried out on DEGs upon HMGA1 silencing in MDA-MB-231 cells to cluster these genes in functions related to angiogenesis. The functions are ranked by *p*-value and the number of genes found to be regulated by HMGA1/function was annotated. **c** Representative immunofluorescence images of capillary-like structures (green) formed on Matrigel by HUVEC cells treated with the supernatant of MDA-MB-231 cells, which have been previously silenced for HMGA1, FOXM1 or HMGA1 and FOXM1 in combination. Serum-free medium and VEGFA were used as negative and positive controls respectively. **d** The number of vessels formed by HUVEC cells were counted and plotted on the graph: the white and the green bars correspond to the negative and positive controls respectively, while the supernatants from control MDA-MB-231 cells or from MDA-MB-231 cells silenced for HMGA1, FOXM1 or co-silenced for HMGA1 and FOXM1 are represented with the black, grey, light blue and purple bars respectively. The data are presented as the mean ± SD (n = 4). *p < 0.05*,* **p < 0.01, ***p < 0.001*;* two-tailed Student’s *t*-test is calculated with respect to siCTRL

Gene Ontology (GO) analysis of our RNA-Seq data revealed that the proteins coded by HMGA1-differentially expressed genes are enriched in exosome-secreted and cell-cell junction terms; this result suggested that HMGA1 regulates secreted or membrane-bound proteins (Fig. 6a). Moreover, we found a significant number of genes associated with GOs related to angiogenesis (Fig. 6b). We, therefore, asked whether HMGA1 and FOXM1 are involved in tumor angiogenesis. To this aim, we treated primary human umbilical vein endothelial cells (HUVEC) (ECs) with conditioned medium derived from MDA-MB-231 silenced for the expression of HMGA1, FOXM1 or the combination of the two factors and we analyzed their proliferation, migration and vessel formation capacity. HUVEC proliferation was assessed analyzing the expression of the proliferation marker Ki-67. We observed that the silencing of HMGA1 and FOXM1 reduced the ability of breast cancer cells-conditioned medium to promote the proliferation of ECs (Additional file 9: Figure S6a). Then, we investigated the impact on ECs migration by assessing the migratory ability of HUVEC cells through transwell membrane inserts coated with fibronectin. ECs were plated on the upper chamber and conditioned medium from MDA-MB-231 cells was added in the lower chamber as angiogenic inducer. We observed that ECs migrated slower under the stimuli of the conditioned medium derived from MDA-MB-231 cells silenced for HMGA1 or FOXM1 (Additional file 9: Figure S6b). Finally, we evaluated the capacity of conditioned medium derived from MDA-MB-231 cells to induce vessel formation using an in vitro tube formation assay. ECs were grown in Matrigel and incubated for 18 h with conditioned medium; then, ECs were stained for actin to visualize tube formation. The conditioned medium from MDA-MB-231 induced the formation of capillary-like tubular structures connected to each other creating a mesh-like structure on the gel, a pattern analogous to the one observed with VEGFA treatment used as positive control (Fig. 6c). In contrast, ECs incubated with conditioned medium from breast cancer cells silenced for HMGA1 and FOXM1 formed aggregates losing the capillary-like structures (Fig. 6c). Interestingly, this outcome is stronger when HMGA1 and FOXM1 were silenced concomitantly, as we can appreciate from the quantitative analysis of the data (Fig. 6d). These results indicate a clear role of HMGA1 and FOXM1 in modulating the tumor angiogenic capacity of breast cancer cells on ECs.

### HMGA1 and FOXM1 modulate the in vivo proangiogenic activity

To further assess the activity of HMGA1 and FOXM1 in regulating vessel formation, we took advantage of zebrafish (*Danio rerio*) embryos as in vivo animal models for angiogenesis studies, that are widely used for their rapid development, optical transparency and because they possess a circulatory system similar to that of mammals [52, 53]. With this aim, we used a zebrafish strain expressing the Enhanced Green Fluorescence Protein (EGFP) in endothelial cells, which allows following tumor neo-angiogenesis. The same number of MDA-MB-231 cells, silenced for HMGA1, FOXM1 and the two factors in combination or with a control siRNA, was injected into the yolk sac of zebrafish embryos. At 1 day post injection (1dpi), we clearly observed the growth of a tumor mass (Additional file 10: Figure S7a) and we quantified the proliferation rate analyzing the expression of the proliferation marker Ki-67. Reduced expression of Ki-67 was observed in the tumor mass of MDA-MB-231 cells silenced for HMGA1 and FOXM1 (Additional file 10: Figure S7c).

**Fig. 7:**
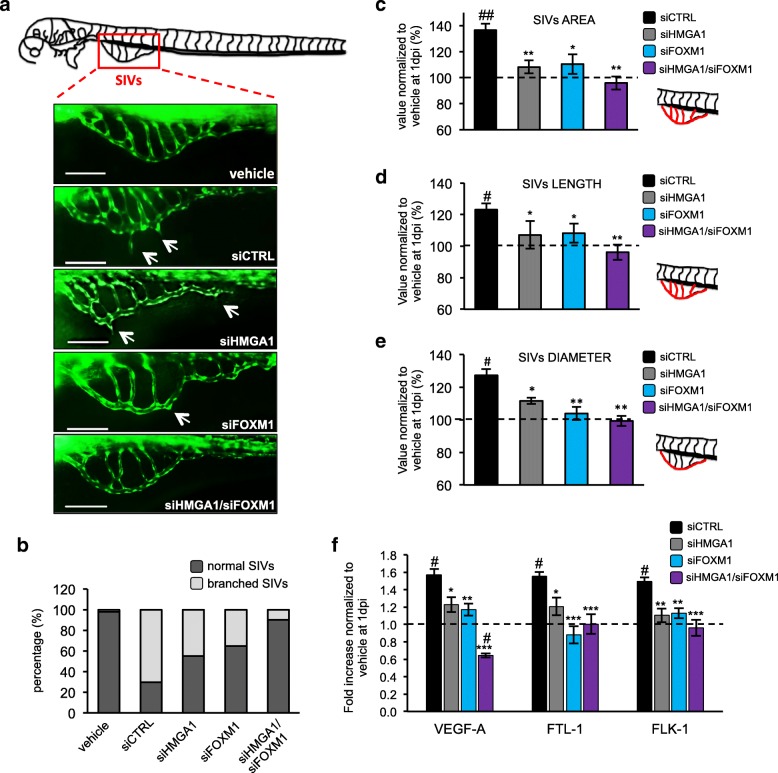
HMGA1 and FOXM1 modulate in vivo the proangiogenic activity. **a** Schematic representation of the sub-intestinal veins (SIVs) evaluated for the assessment of the zebrafish angiogenic process. Below, representative images of SIVs (green) taken at 1 day post injection (dpi) of MDA-MB-231 cells silenced for HMGA1 and/or FOXM1 or control cells on zebrafish Tg (fli1:EGFP)Y1 embryos. The white arrows indicate an alteration of the SIVs. The culture medium was used as a negative control. Bar = 100 μm. **b** Embryos with a branched SIVs (light grey) or unaltered SIVs (dark grey) were counted and plotted on the graph, expressed in percentage (*n = 30*). The area (**c**), length (**d**) and diameter (**e**) of the SIVs (schematic representation of the vessels considered for each parameter is reported on the right of the graphs and coloured in red) were measured in zebrafish embryos at 1 dpi of MDA-MB-231 cells silenced for HMGA1 (grey bar), FOXM1 (light blue bar) or co-silenced for the two factors (purple bar). The data are normalized to control (vehicle) represented as a black dotted line and are represented as the mean ± SD (*n = 30).* ^*#*^*p* < 0.05, ^*##*^*p* < 0.01 respect to the vehicle. *p < 0.05, **p < 0.01 respect to the siCTRL. **f** qRT-PCR analysis of zebrafish VEGFA, FLT1 and FLK1 at 1 dpi in embryos injected with control MDA-MB-231 (black bar) or MDA-MB-231 cells silenced for HMGA1 (grey bar), FOXM1 (light blue bar) or co-silenced for the two factors (purple bar). Data were normalized to the zebrafish β-Actin mRNA amount. The data are compared to the control (vehicle) and are represented as the mean ± SD (*n = 60*). ^*#*^*p* < 0.05 respect to the vehicle. *p < 0.05, **p < 0.01 and ***p < 0.001 respect to the siCTRL

Next, we examined the neovascularization of the sub-intestinal veins (SIVs) at 1dpi (Fig. 7a and b); embryos injected with medium without cells (vehicle) were used as control normal condition. We firstly evaluated the SIVs morphology in our experimental conditions and we classified the animals as with “normal SIVs” or “branched SIVs” with respect to the absence/presence of ectopic processes from the vessels in the yolk (Fig. 7b). The quantitative analysis showed a strong induction of branched SIVs after injection of MDA-MB-231 control cells, whereas cells silenced for HMGA1 and FOXM1 reduced the number of animals with branched SIVs. Interestingly, the simultaneous depletion of HMGA1 and FOXM1 enable to almost complete abolish the alteration in the neovascularization (Fig. 7b). To better define these results we quantified the area, length and diameter of the SIVs. For all the parameters analyzed we found that breast cancer cells silenced for HMGA1 and FOXM1 reduced these parameters and, moreover, the concomitant silencing of the two factors restored normal conditions (Fig. 7c-e). Strikingly, the analysis of mRNA of zebrafish angiogenesis markers (zVEGF-A, zFTL-1 and zFLK-1) correlates with the observed tumor neo-angiogenesis. In fact, zVEGF-A, zFTL-1 and zFLK-1 expression levels were increased in animals injected with control cells, whereas in embryos with cells silenced for HMGA1 and FOXM1 their levels were comparable to normal levels (Fig. 7f). Our data demonstrate a synergic role of HMGA1 and FOXM1 in governing tumor angiogenesis using an in vivo model.

### HMGA1, FOXM1 and VEGFA co-expression is associated with a poor prognosis in breast cancer patients

**Fig. 8:**
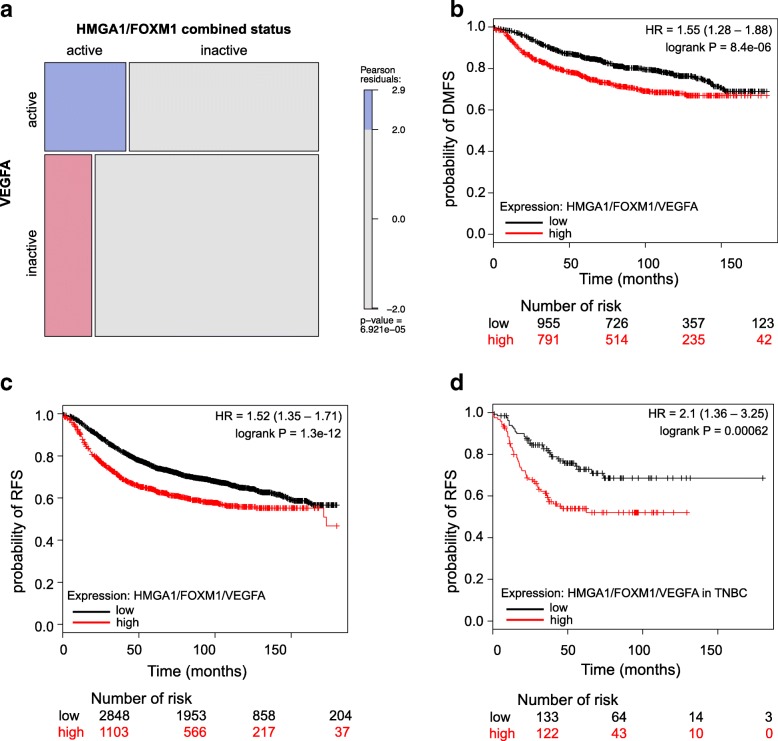
HMGA1, FOXM1 and VEGFA co-expression is associated with a poor prognosis in breast cancer patients. **a** Mosaic plot showing the proportion of patients stratified by VEGFA expression and HMGA1/FOXM1 activation. Color indicates Pearson residuals. **b** Kaplan Meier curve of DMFS (distant metastasis-free survival) in a cohort of breast cancer patients stratified by HMGA1/FOXM1/VEGFA expression. **c** and **d** Kaplan Meier curves of RFS (relapse-free survival) in a cohort of breast cancer (**c**) and TNBC (**d**) patients stratified by HMGA1/FOXM1/VEGFA expression

To assess whether our findings could be related to the pathogenesis of human breast cancer, we interrogated public available datasets in the TCGA repository. Firstly, we investigated the relationship between the combined expression of HMGA1 and FOXM1 with the VEGFA expression in breast cancer patients. Thus, we stratified breast cancer samples according to their relative expression levels of HMGA1 and FOXM1, obtaining a significant enrichment in VEGFA expression in patients in which the HMGA1/FOXM1 axis is activated (Fig. 8a). Furthermore, in gene-expression datasets of breast cancer samples we observed that patients with high levels of HMGA1, FOXM1 or VEGFA had a statistically significant lower survival probability in terms of distant metastasis-free survival and relapse-free survival with respect to HMGA1, FOXM1 and VEGFA low expression patient groups (Additional file 11: Figure S8). Interestingly, the low survival probability of patients that co-express HMGA1, FOXM1 and VEGFA at high levels has also been observed both in breast cancer datasets and in a subset of TNBC patients (Fig. 8b-d). Overall, clinical datasets analysis confirmed that VEGFA expression positively correlates with HMGA1 and FOXM1, and that their presence has an impact on breast cancer development, since a signature combining HMGA1, FOXM1 and VEGFA expression is associated to a worse prognosis.

## Discussion

HMGA1 is an architectural transcription factor, widely considered a master regulator of breast cancer progression [7, 13, 17, 54]. Indeed, it has a causal role, both at early stages, bringing the mammary epithelial cells to acquire a malignant phenotype [55], and during breast cancer progression, by promoting cellular migration and invasion capabilities, consequently leading to the metastatization event [13, 17]. Thus, deepening the knowledge about the molecular mechanisms that HMGA1 controls will be paramount for the discovery of new targeted and efficacious therapies. With the aim to identify new molecular partners of HMGA1 in regulating breast cancer gene networks, we performed RNA-Seq analysis of TNBC cells depleted for HMGA1 expression. Here, we have discovered FOXM1 as a novel HMGA1-molecular partner, demonstrating that HMGA1 stabilizes FOXM1 in the nucleus preventing its degradation, increasing FOXM1-dependent transcriptional activity and potentiating its crucial role in tumour angiogenesis.

It is known that HMGA1 interacts with several transcription factors and guides their action on a high number of target genes involved in many cellular processes such as cell growth, proliferation, differentiation and cell death [7]. We found that our bioinformatic analysis on RNA-Seq data from TNBC cells silenced for HMGA1 is very robust in discovering HMGA1-molecular partners. In fact, among the putative partners obtained by our analysis, we found RB1 and E2F, whose connection with HMGA family members has been previously described. Specifically, HMGA1 enhances E2F transcriptional activity by directly binding RB1, inhibiting its tumor suppressive activity [40]. Our data indicate that HMGA1 could modulate the activity of these factors also in breast cancer progression, bringing to light common crucial HMGA1-oncogenic pathways in different cancer types. Moreover we found FOXM1, whose pathway is the top up-regulated in TNBC [23], as HMGA1 partner. We demonstrate that HMGA1 and FOXM1 regulate a common gene network, characterized by factors with a clear role in cancer EMT, migration, and angiogenesis, key processes involved in conferring aggressiveness to TNBC. Among the genes, CCNE2, which has been connected to the migratory ability of tumoral cells [56], has been proved to be under the transcriptional control of HMGA1, thus promoting the migratory and invasive abilities of breast cancer cells [18]. Furthermore, HMGA1 has been found to regulate the EMT, a process necessary to cell migration, by impacting on the WNT-beta catenin pathway, known to contribute to metastatization [57]; indeed, HMGA1 regulates LEF1, one of the actors of this pathway [13]. A considerable amount of data sustains the involvement of FOXM1 in several features of breast cancer progression, such as the EMT, the migration and invasion abilities of tumor cells, in which FOXM1 controls the transcription of several metalloproteinases and the EMT inducer SNAI2 [58]. Our results demonstrate that HMGA1 cooperates with FOXM1 in regulating the expression of a common gene network, enhancing the aggressiveness of TNBC cells, highlighting a dependence of breast tumor cells on HMGA1 and FOXM1 synergic action.

We observed that HMGA1 forms a complex with FOXM1 and improves FOXM1 protein stability and nuclear localization, while in the absence of HMGA1, FOXM1 is led to proteasomal degradation. These findings suggest that HMGA1, by stabilizing FOXM1, increases its transcriptional activity. This is consistent with literature data showing that FOXM1 is tightly regulated by its molecular partners that can increase FOXM1 transcriptional activity by promoting its stabilization and nuclear localization [42, 59, 60], as for instance it has been shown for the interaction with MTDH in glioblastoma [61].

Angiogenesis is one of the cancer hallmarks in which FOXM1 is mainly involved, inducing matrix metalloproteinase genes as well as VEGFA [62]. As VEGFA is a growth factor essential for normal and pathological angiogenesis, its gene is finely regulated by a plethora of transcription factors, such as Sp1/Sp3, AP-2, Egr-1, STAT3 and HIF1, integrating multiple signals [44]. In particular, it has been demonstrated that HMGA1 regulates VEGFA gene expression in diabetic retinopathy and, by interacting with HIF1, in 3T3 L1 adipocytes [45, 63]. Intriguingly, in this study we found that HMGA1 regulates the transcription of VEGFA, acting through two independent ways: on one side, HMGA1 potentiates the transcriptional activity of FOXM1; on the other, HMGA1 acts through Sp1 in a different promoter region. Consistently, the observation that HMGA1 interacts with Sp1 is also reported in the control of insulin receptor (IR) gene transcription, in which HMGA1, by interacting with Sp1 and C/EBP beta, facilitates the binding of both factors to the IR promoter, synergistically activating IR transcription [51]. Similarly, HMGA1 by interacting with FOXM1 could enhance its binding to the VEGFA promoter. Our results show that HMGA1, by preventing FOXM1 degradation, increases its level and therefore this could account for the increase of FOXM1 transcriptional activity. We cannot exclude that this result is attributable to one hypothesis or the other, or to the combination of both.

Our data clearly show that HMGA1 and FOXM1, in addition to regulating VEGFA, have a strong impact on tumor angiogenesis. Previous works performed in a rat model of cerebral ischemia and in HUVEC cells showed an involvement of HMGA1 in modulating angiogenic proteins, such as Angiopoietin-1, a factor fundamental in maintaining the tumor vascularization [64, 65]. Our data demonstrate for the first time a direct involvement of HMGA1 in the process of tumor angiogenesis. Indeed, the supernatants of TNBC cells depleted for HMGA1 and FOXM1 abolished the ability of endothelial cells to organize in vessel-like structures. In line with this aspect, we observed that MDA-MB-231 cells injected in zebrafish embryos induced abnormalities in angiogenesis of the host and that this phenotype is abolished when HMGA1 and FOXM1 are depleted. These findings suggest that HMGA1 and FOXM1 have a leading role in guiding breast cancer cells to secrete pro-angiogenic factors. In accordance to this, we previously demonstrated that HMGA1 has a profound impact in the breast cancer cell secretome, inducing the release of a pool of pro-migratory proteins that act with an autocrine mechanism [20]. This study provides an additional explanation of the mechanism of HMGA1 in promoting TNBC aggressiveness. Indeed, in breast cancer patients a profound molecular relation between HMGA1, FOXM1 and VEGFA has been further highlighted by TCGA analysis, which confirmed a strong enrichment of VEGFA in patients that overexpressed HMGA1 and FOXM1. Moreover, the co-expression of the three factors has been found to be a poor prognostic value of DMFS and RFS in breast cancer patients.

## Conclusions

Our results provide further evidence on molecular signals governing breast tumor angiogenesis under the coordinate control of the two master regulators of tumorigenesis HMGA1 and FOXM1. Drugs targeting FOXM1 such as thiostrepton [66, 67] and FDI-6 [68] and HMGA such as trabectedin [69, 70] have already been reported; therefore the possibility to target HMGA1 and FOXM1 in combination should represent an attractive therapeutic option in tumors expressing both factors.

## Additional files

Additional file 1: **Table S1.** List of primers used in qRT-PCR analyses. (PDF 44 kb)

Additional file 2: **Figure S1.** Clustering of gene expression data showing expression levels at different time points before and after silencing of HMGA1**.** Color intensity corresponds to the row Z-Score. (PDF 1107 kb)

Additional file 3: **Tables S2.** and **S3.** GSEA analysis on 24 and 72 h siHMGA1-regulated genes. (PDF 72 kb)

Additional file 4: **Table S4.** List of differentially expressed genes after HMGA1-silencing at 24 (a) and 72 (b) hours in common with FOXM1-gene network. (PDF 35 kb)

Additional file 5: **Figure S2.** (**a**) Boxplots showing the expression levels of FOXM1 mRNA in breast cancer samples with high and low expression of HMGA1 (**b**) Schematic representation of the pGL3-5BS reporter vector. The 5 binding elements of FOXM1 are represented with white boxes. (**c**) Western blot analysis of HEK293T transfected with pEGFP-FOXM1 (600 ng) and increasing amounts (200, 400 and 600 ng) of pEGFP-HMGA1, using an α-GFP as primary antibody. pRL-CMV Renilla luciferase expression vector was used to normalize for transfection efficiencies. (**d**) Confirmation of gene silencing. qRT-PCR of HMGA1 and FOXM1 levels after 72 h of HMGA1 (grey bar), FOXM1 (light blue bar) and HMGA1/FOXM1 (purple bar) silencing in MDA-MB-231 cell line. GAPDH was used for normalization. The data are compared to siCTRL and are presented as the mean ± SD (*n* = 3), ***p < 0.001; two-tailed Student’s *t*-test. (**e**) qRT-PCR analyses of selected HMGA1/FOXM1 target genes (CCNE2, LEF1 and VEGFA) in MDA-MB-231 cells silenced for HMGA1 (grey bar), FOXM1 (light blue bar) and HMGA1/FOXM1 (purple bar) at 72 h. GAPDH was used for normalization. The data are compared to siCTRL and are presented as the mean ± SD (*n* = 3), *p < 0.05, **p < 0.01, ***p < 0.001; two-tailed Student’s *t*-test. (**f**) Western blot validations of HMGA1 and/or FOXM1 silencing in wound closure assays in MDA-MB-231 (left) and MDA-MB-157 (right) cell lines are reported. β-actin was used as a loading control. (PDF 3217 kb)

Additional file 6: **Figure S3.** (**a**) Representative immunofluorescence images of the translocation of FOXM1 (green) and its colocalization with the Subunit β5 of the proteasome (red) in MDA-MB-231 control cells versus cells silenced for HMGA1. Images were taken at 60X magnification. (**b**) and (**c**) Representative immunofluorescence images of the translocation of FOXM1 (green) and its colocalization with the Subunit β2 (**b**) and β5 (**c**) of the proteasome (red) in MDA-MB-157 control cells versus cells silenced for HMGA1. Images were taken at 60X magnification. (**d**) Representative images of FOXM1-GFP after HMGA1 silencing and pEGFP-FOXM1 transfection in MDA-MB-231 cells. White arrows indicate the translocation of FOXM1 after HMGA1 silencing. (**e**) Representative images of FOXM1-GFP after HMGA1 silencing and pEGFP-FOXM1 transfection in HEK293T cells. White arrows indicate the cellular localization of FOXM1 after HMGA1 silencing. (PDF 1197 kb)

Additional file 7: **Figure S4.** (**a**) Boxplots showing the expression levels of VEGFA mRNA in breast cancer samples. The samples were stratified based on HMGA1 (left) and FOXM1 (right) mRNA expression levels. (**b**) and (**c**) Luciferase assays on HEK293T cells transiently co-transfected with the luciferase reporter plasmid pGL4.10-VEGFprom (− 1000–1) with increasing quantities of either the expression plasmid pEGFP-FOXM1 (b) or pEGFP-HMGA1 (c). pRL-CMV Renilla luciferase expression vector was included to normalize for transfection efficiencies. Values are reported as relative luciferase activity comparing to cells transfected with the expression control vector pEGFP. The data are represented as the mean ± SD (*n =* 3). **p < 0.01, ***p < 0.001; two-tailed Student’s *t*-test. On the right, the correspondent western blot validation is reported. (PDF 507 kb)

Additional file 8: **Figure S5.** (**a**) Schematic representation of the bioinformatic analysis of the 1000 bp VEGFA promoter sequence cloned upstream the luciferase sequence in pGL4.10-VEGFprom (− 1000–1) used in reporter experiments. FOXM1 binding sites are represented with light blue ovals, whereas the AT-enriched sequences bound by HMGA1 are figured as grey boxes. In detailed, we found several AT-rich sequences in the region from – 979 to 907 bp, from − 641 to − 521 bp, from − 355 to − 322 bp, from − 169 to − 75 bp, where the AT stretches are particularly long, and from − 17 to − 13 bp from the TSS of the VEGFA promoter. In addition, we found 9 putative FOXM1 binding sites from − 993 to − 922 bp, from − 643 to – 638 bp, from − 326 to − 322 bp, from − 124 to − 104 bp, where it is located the FOXM1 preferential binding sequence TAAACA, and from − 17 to − 13 bp from the TSS of the VEGFA promoter. (**b**) Schematic representation of deletion reporter vectors pGL4.10-VEGF (− 1000–500) and pGL4.10-VEGF (− 500–1) obtained from pGL4.10-VEGFprom (− 1000–1). (**c**) Luciferase assay on HEK293T cells transiently co-transfected with the luciferase reporter plasmid pVEGFprom (− 1000–1), the deletion mutants pVEGFprom (− 1000–500) or pVEGFprom (− 500–1) with the expression plasmids pEGFP-HMGA1 and pEGFP-FOXM1. pRL-CMV Renilla luciferase expression vector was included to normalize for transfection efficiencies. Values are reported as relative luciferase activity comparing to cells transfected with the expression plasmid pEGFP. The data are represented as the mean ± SD (*n* = 3). *NS*: not significant; two-tailed Student’s *t*-test. An example of western blot validations is reported. (**d**) and (**e**) Representative images of western blot validations of experiments presented in Fig. 5b and d respectively. (PDF 2000 kb)

Additional file 9: **Figure S6.** HUVEC cells were treated with MDA-MB-231 cells supernatants, who had been previously silenced for HMGA1, FOXM1 or co-silenced for HMGA1 and FOXM1. Serum-free medium and normal human serum (NHS) were used as negative and positive controls respectively. (**a**) The proliferation of HUVEC cells was investigated by the positivity to the Ki67 marker and expressed in terms of relative % of fluorescence respect to negative control (CTRL-). The data are represented as the mean ± SD (*n* > 3); **p < 0.001, ***p < 0.001; two-tailed Student’s *t*-test. (**b**) The migration of endothelial cells was assessed by Transwell assay, adding the supernatants of MDA-MB-231 in the lower chambers of the multiwell. The number of cells migrated were counted and the results are expressed as relative % of migration, respect to negative control (CTRL-). The data are represented as the mean ± SD (*n > 3*). *p < 0.05; two-tailed Student’s *t*-test. (**c**) Representative western blot validations of HMGA1/FOXM1 silencing in MDA-MB-231 cells used to collect supernatants are reported. β-actin was used as a loading control. (PDF 704 kb)

Additional file 10: **Figure S7.** (**a**) Representative live images of the tumor masses (red) at 1dpi in zebrafish Tg (fli1:EGFP)^Y1^ embryos microinjected with MDA-MB-231 cells pre-treated with siCTRL, siHMGA1, siFOXM1 or siHMGA1/siFOXM1. Just before the microinjection, tumor cells (red) were stained with the fluorescent DiI stain. Scale bar = 100 μm. (**b**) A representative western blot validation of HMGA1/FOXM1 silencing in MDA-MB-231 cells microinjected in zebrafish embryos is reported. β-actin was used as a loading control. (**c**) qRT-PCR analysis of human Ki67 at 1 dpi in control MDA-MB-231 cells (black bar) and MDA-MB-231 cells silenced for HMGA1 (grey bar), FOXM1 (light blue bar) or cosilenced for the two factors (purple bar). Data were normalized to the human GAPDH mRNA amount. The data are represented as the mean ± SD (*n =* 60*).* *p < 0.05, **p < 0.01, ***p < 0.001**;** two-tailed Student’s *t*-test. (PDF 2803 kb)

Additional file 11: **Figure S8.** (**a**-**c**) Kaplan Meier curves of DMFS in a cohort of breast cancer patients stratified by HMGA1 (**a**), FOXM1 (**b**) and VEGFA (**c**) expression. (**d**-**f**) Kaplan Meier curves of RFS in a cohort of breast cancer patients stratified by HMGA1 (**d**), FOXM1 (**e**) and VEGFA (**f**) expression. (PDF 387 kb)

## Data Availability

The RNA-Seq data generated during the current study are available in the NCBI Gene Expression Omnibus (GEO; https://www.ncbi.nlm.nih.gov/geo/) repository, under accession number GSE129915.

## Abbreviations

CM: Conditioned Medium
Co-IP: Co-immunoprecipitation
DEGs: Differentially expressed genes
DMFS: Distant metastasis-free survival
EC: Endothelial cells
EGFP: Enhanced Green Fluorescence Protein
EMT: Epithelial to mesenchymal transition
FOXM1: Forkhead box M1
GEO: Gene Expression Omnibus
GO: Gene Ontology
GSEA: Gene Set Enrichment Analysis
HMGA1: High Mobility Group A1
HUVEC: Human umbilical vein endothelial cells
IPA: Ingenuity Pathway Analysis
NHS: Normal Human Serum
qRT-PCR: quantitative Reverse Transcription Polymerase Chain Reaction
RFS: Relapse-free survival
siRNA: small interfering RNA
TCGA: The Cancer Genome Atlas
TF: Transcription factor
TNBC: Triple-negative breast cancer
VEGFA: Vascular Endothelial Growth Factor
