# Supplementary material for: HMGA1 promotes breast cancer angiogenesis supporting the stability, nuclear localization and transcriptional activity of FOXM1
Source: J Exp Clin Cancer Res. 2019 Jul 16;38:313. doi: 10.1186/s13046-019-1307-8 (PMC6636010; doi:10.1186/s13046-019-1307-8)
Supplement: Supplementary file 1 — Table S1. List of primers used in qRT-PCR analyses. (PDF 44 kb) [file 13046_2019_1307_MOESM1_ESM.pdf]

Additional file 1: Table S1.

Target gene/ primer name	Forward primer (5'- 3')	Reverse Primer (5'- 3')
hGAPDH	TCTCTGCTCCTCCTGTTC	GCCCAATACGACCAAATCC
hHMGA1	ACCAGCGCCAAATGTTCATCCTCA	AGCCCCTCTTCCCCACAAAGAGT
hFOXM1	AGAAACGACCGAATCCAGAGC	CTGACCCGTGGTAGCAGTG
hVEGFA	AGGAGGGCAGAATCATCACG	ACCAGGGTCTCGATTGGATG
hCCNE2	TGAGCCGAGCGGTAGCTGGT	GGGCTGGGGCTGCTGCTTAG
hLEF1	CGAATGTCGTTGCTGAGTGT	GCTGTCTTTCTTTCCGTGCT
zVEGFA (Zebrafish VEGFA)	GATGTGATTCCCTTCATGGATGTGT	GGATACTCCTGGATGATGTCTACCA
zFLT-1 (Zebrafish VEGF receptor 1)	AACTCACAGACCAGTGAACAAGATC	GCCCTGTAACGTGTGCACTAAA
zFLK-1 (Zebrafish VEGF receptor 2	GACCATAAAACAAGTGAGGCAGAAG	CTCCTGGTTTGACAGAGCGATA
zACTIN (Zebrafish actin)	CAGCAAGCAGGAGTACGATGAGT	TTGAATCTCATTGCTAGGCCATT
hKi67 (human Ki67)	GCCTGCTCGACCCTACAGA	GCTTGTCAACTGCGGTTGC
hGAPDH (human GAPDH)	CCCATCACCATCTTCCAGGAG	CTTCTCCATGGTGGTGAAGACG
