# Supplementary figures and images for: HMGA1 promotes breast cancer angiogenesis supporting the stability, nuclear localization and transcriptional activity of FOXM1
Source: J Exp Clin Cancer Res. 2019 Jul 16;38:313. doi: 10.1186/s13046-019-1307-8 (PMC6636010; doi:10.1186/s13046-019-1307-8)

Additional file 2: Figure S1.

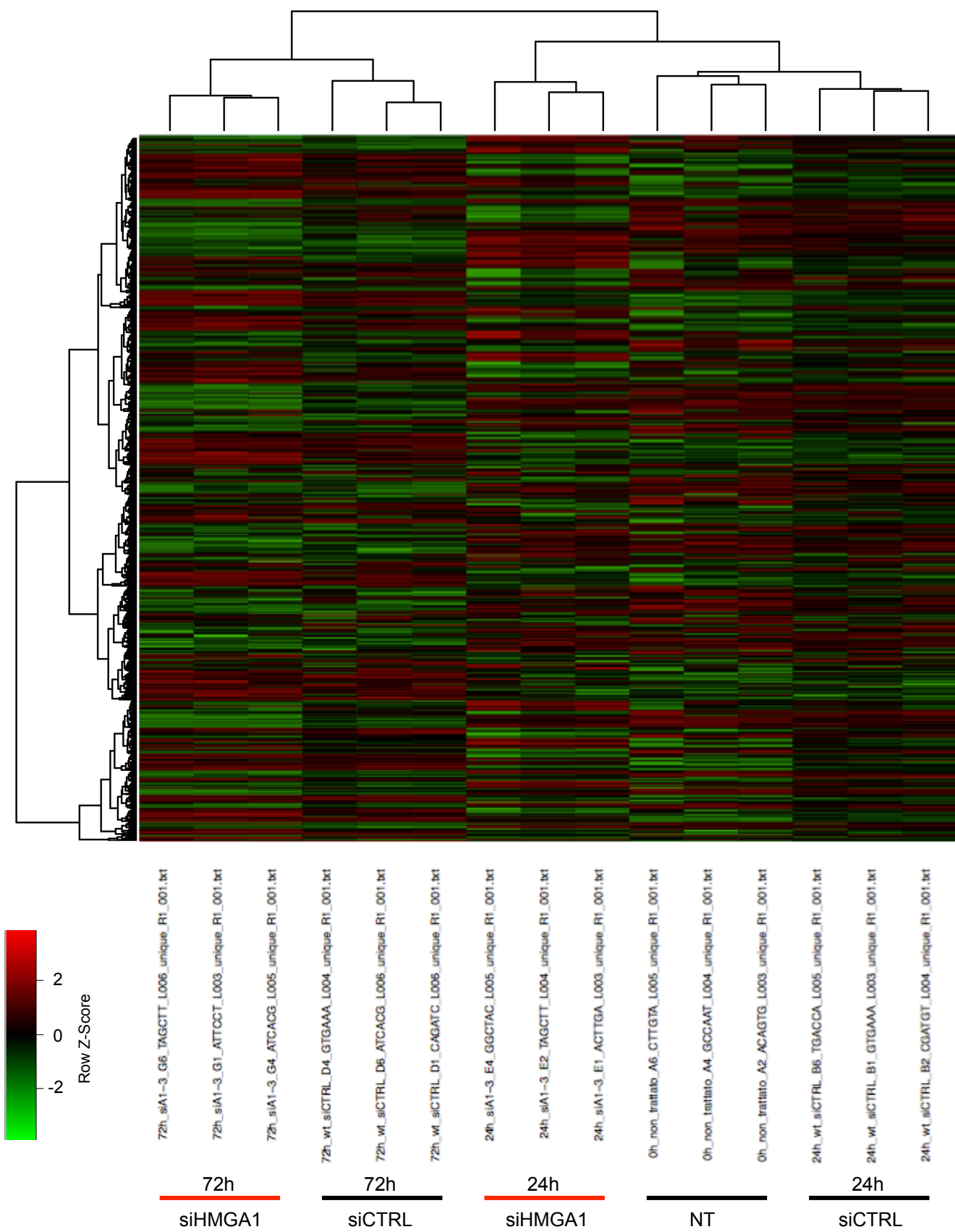

Supplement: Supplementary file 2 — Figure S1. Clustering of gene expression data showing expression levels at different time points before and after silencing of HMGA1. Color intensity corresponds to the row Z-Score. (PDF 1107 kb) [file 13046_2019_1307_MOESM2_ESM.pdf]

Additional file 5: Figure S2.

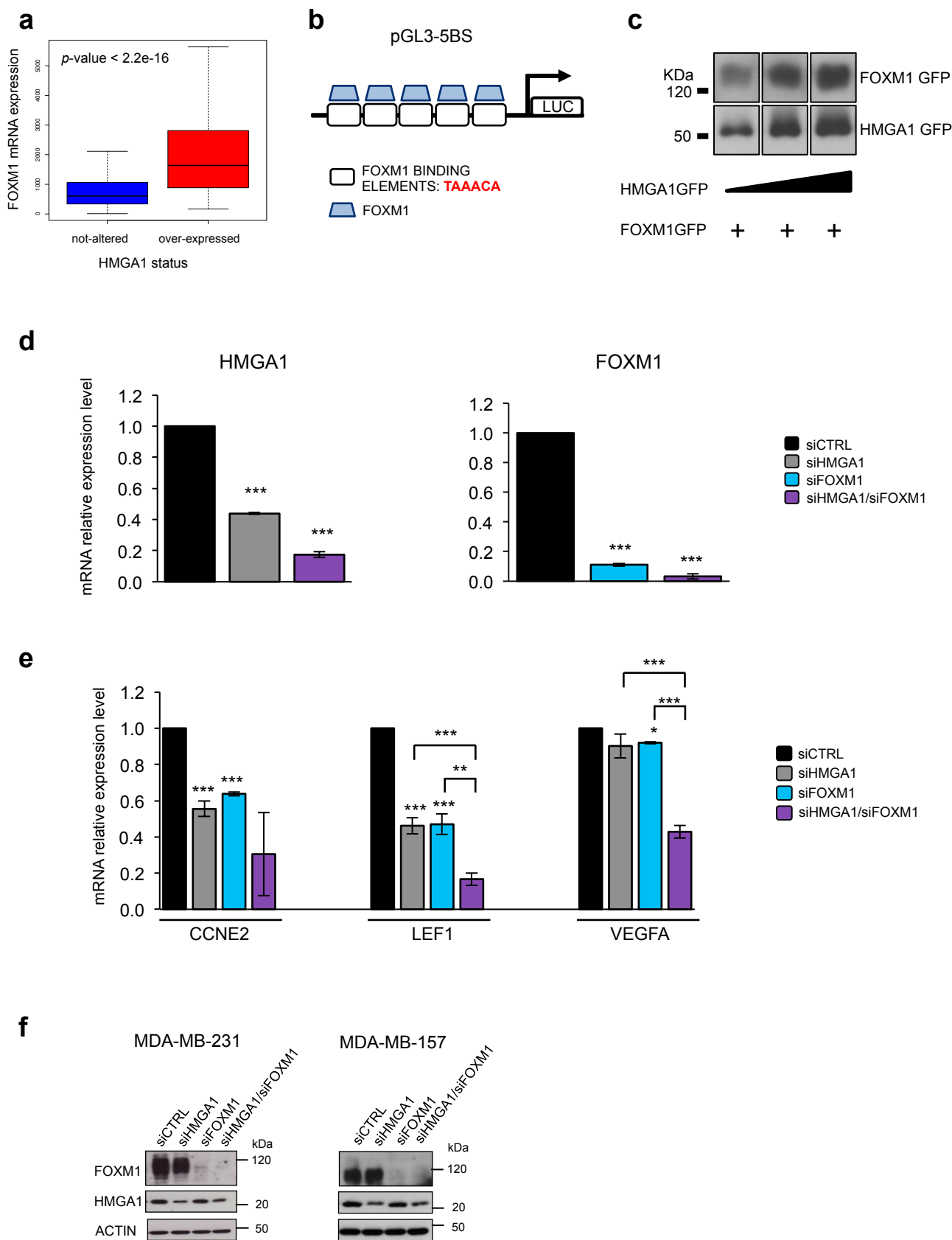

Supplement: Supplementary file 5 — Figure S2. (a) Boxplots showing the expression levels of FOXM1 mRNA in breast cancer samples with high and low expression of HMGA1 (b) Schematic representation of the pGL3-5BS reporter vector. The 5 binding elements of FOXM1 are represented with white boxes. (c) Western blot analysis of HEK293T transfected with pEGFP-FOXM1 (600 ng) and increasing amounts (200, 400 and 600 ng) of pEGFP-HMGA1, using an α-GFP as primary antibody. pRL-CMV Renilla luciferase expression vector was used to normalize for transfection efficiencies. (d) Confirmation of gene silencing. qRT-PCR of HMGA1 and FOXM1 levels after 72 h of HMGA1 (grey bar), FOXM1 (light blue bar) and HMGA1/FOXM1 (purple bar) silencing in MDA-MB-231 cell line. GAPDH was used for normalization. The data are compared to siCTRL and are presented as the mean ± SD (n = 3), ***p < 0.001; two-tailed Student’s t-test. (e) qRT-PCR analyses of selected HMGA1/FOXM1 target genes (CCNE2, LEF1 and VEGFA) in MDA-MB-231 cells silenced for HMGA1 (grey bar), FOXM1 (light blue bar) and HMGA1/FOXM1 (purple bar) at 72 h. GAPDH was used for normalization. The data are compared to siCTRL and are presented as the mean ± SD (n = 3), *p < 0.05, **p < 0.01, ***p < 0.001; two-tailed Student’s t-test. (f) Western blot validations of HMGA1 and/or FOXM1 silencing in wound closure assays in MDA-MB-231 (left) and MDA-MB-157 (right) cell lines are reported. β-actin was used as a loading control. (PDF 3217 kb) [file 13046_2019_1307_MOESM5_ESM.pdf]

Additional file 6: Figure S3.

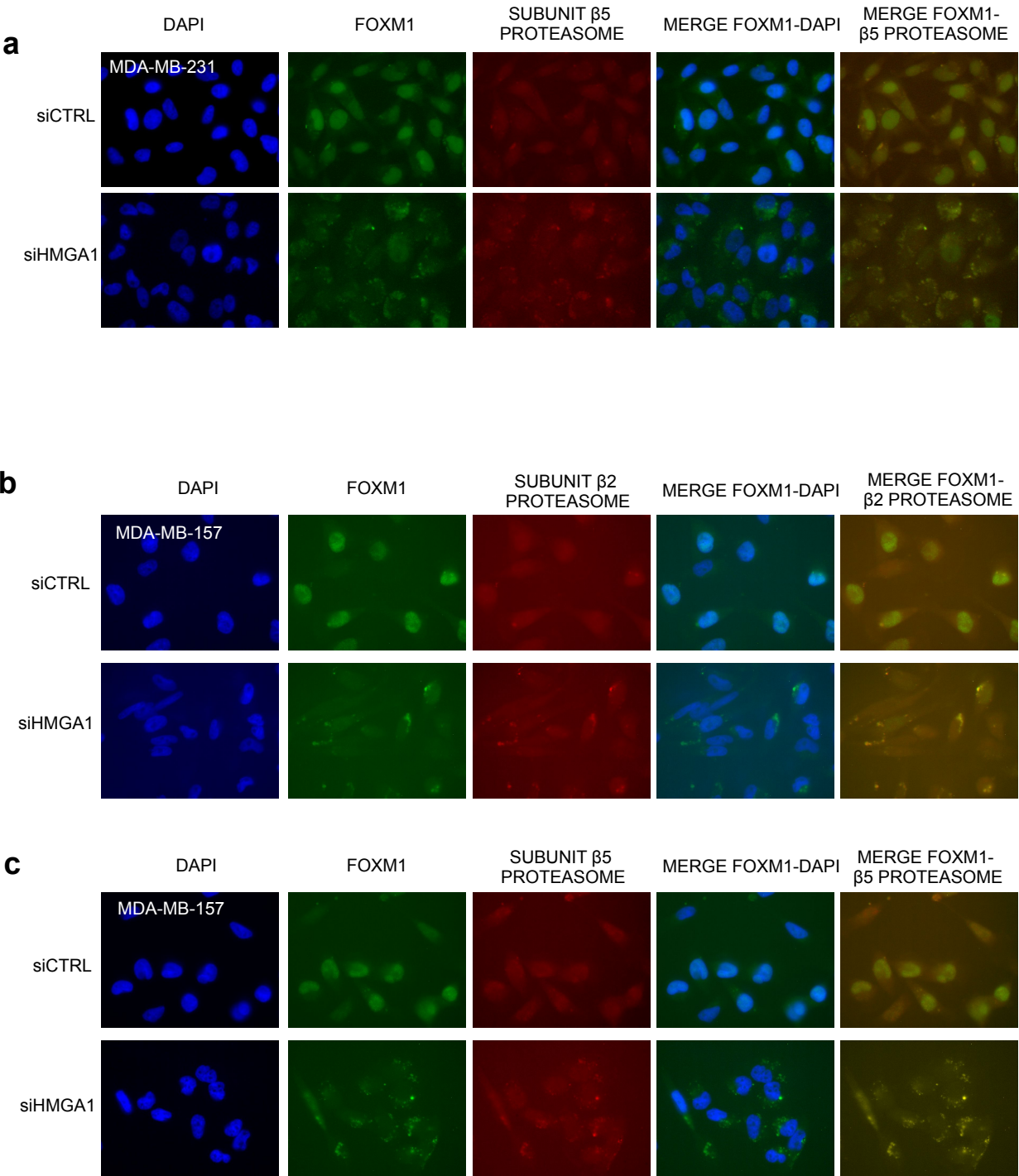

**d**

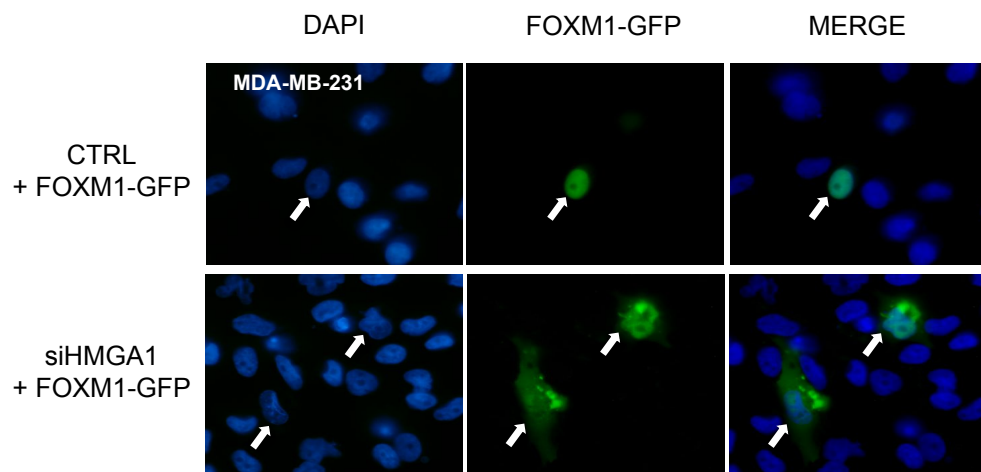

**e**

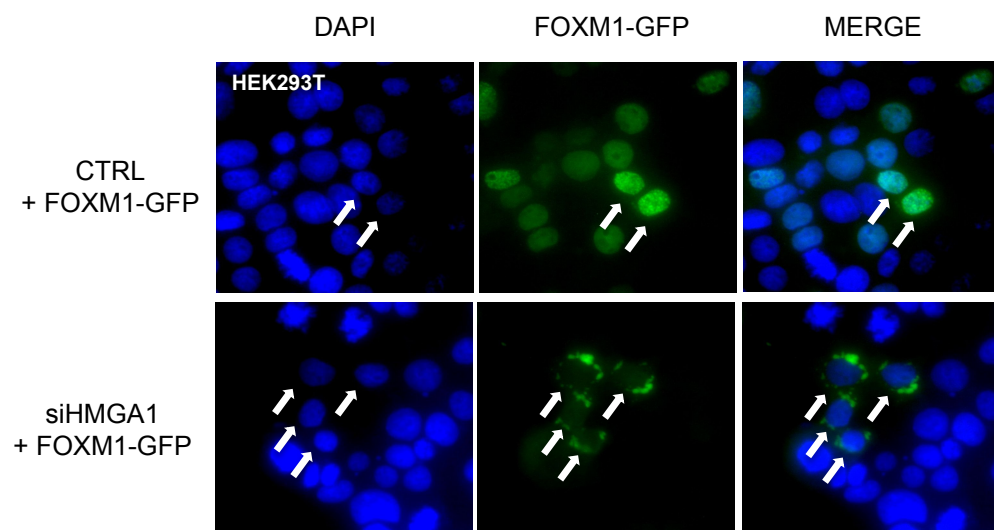

Supplement: Supplementary file 6 — Figure S3. (a) Representative immunofluorescence images of the translocation of FOXM1 (green) and its colocalization with the Subunit β5 of the proteasome (red) in MDA-MB-231 control cells versus cells silenced for HMGA1. Images were taken at 60X magnification. (b) and (c) Representative immunofluorescence images of the translocation of FOXM1 (green) and its colocalization with the Subunit β2 (b) and β5 (c) of the proteasome (red) in MDA-MB-157 control cells versus cells silenced for HMGA1. Images were taken at 60X magnification. (d) Representative images of FOXM1-GFP after HMGA1 silencing and pEGFP-FOXM1 transfection in MDA-MB-231 cells. White arrows indicate the translocation of FOXM1 after HMGA1 silencing. (e) Representative images of FOXM1-GFP after HMGA1 silencing and pEGFP-FOXM1 transfection in HEK293T cells. White arrows indicate the cellular localization of FOXM1 after HMGA1 silencing. (PDF 1197 kb) [file 13046_2019_1307_MOESM6_ESM.pdf]

Additional file 7: Figure S4.

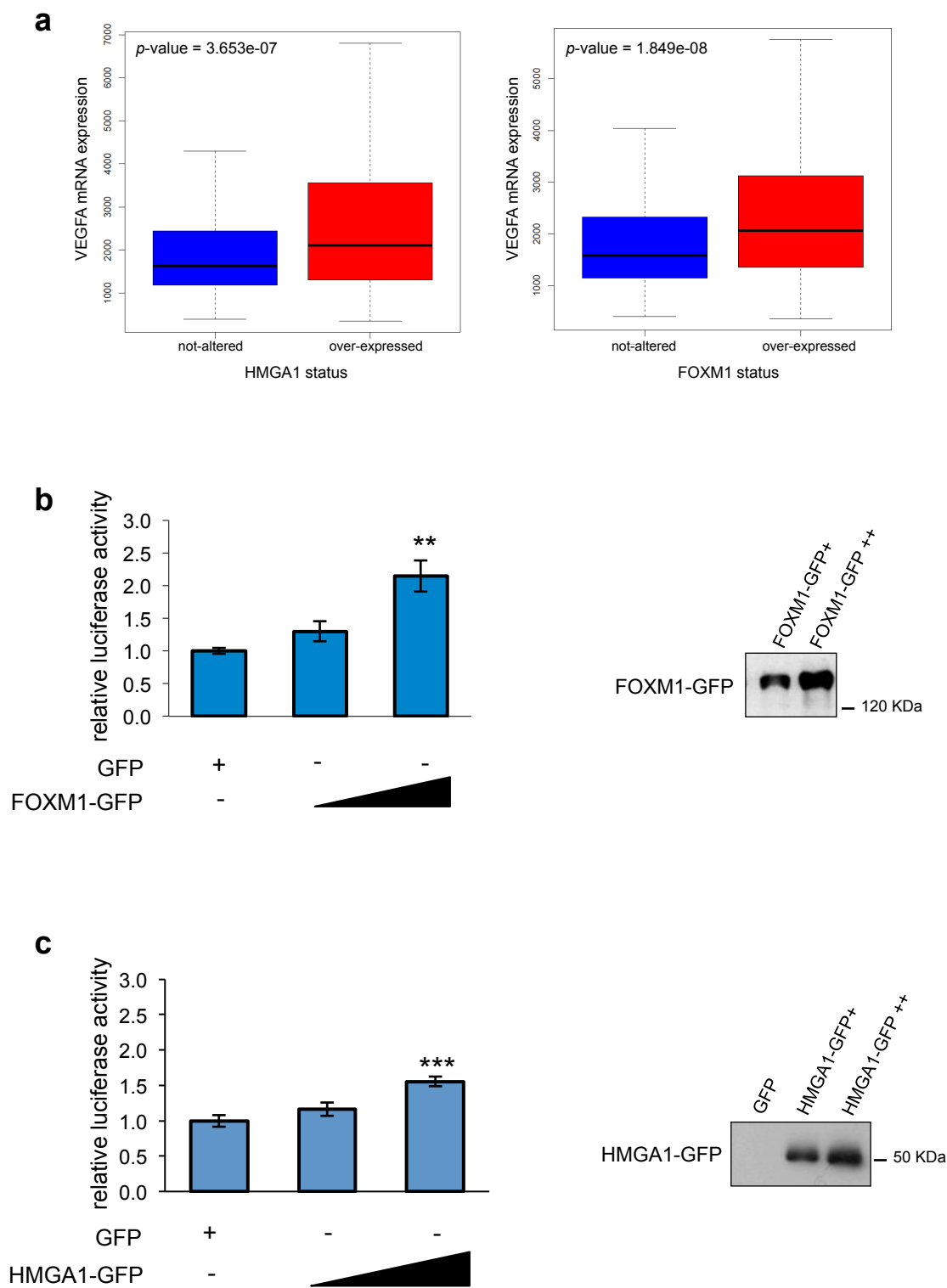

Supplement: Supplementary file 7 — Figure S4. (a) Boxplots showing the expression levels of VEGFA mRNA in breast cancer samples. The samples were stratified based on HMGA1 (left) and FOXM1 (right) mRNA expression levels. (b) and (c) Luciferase assays on HEK293T cells transiently co-transfected with the luciferase reporter plasmid pGL4.10-VEGFprom (− 1000–1) with increasing quantities of either the expression plasmid pEGFP-FOXM1 (b) or pEGFP-HMGA1 (c). pRL-CMV Renilla luciferase expression vector was included to normalize for transfection efficiencies. Values are reported as relative luciferase activity comparing to cells transfected with the expression control vector pEGFP. The data are represented as the mean ± SD (n = 3). **p < 0.01, ***p < 0.001; two-tailed Student’s t-test. On the right, the correspondent western blot validation is reported. (PDF 507 kb) [file 13046_2019_1307_MOESM7_ESM.pdf]

Additional file 8: Figure S5.

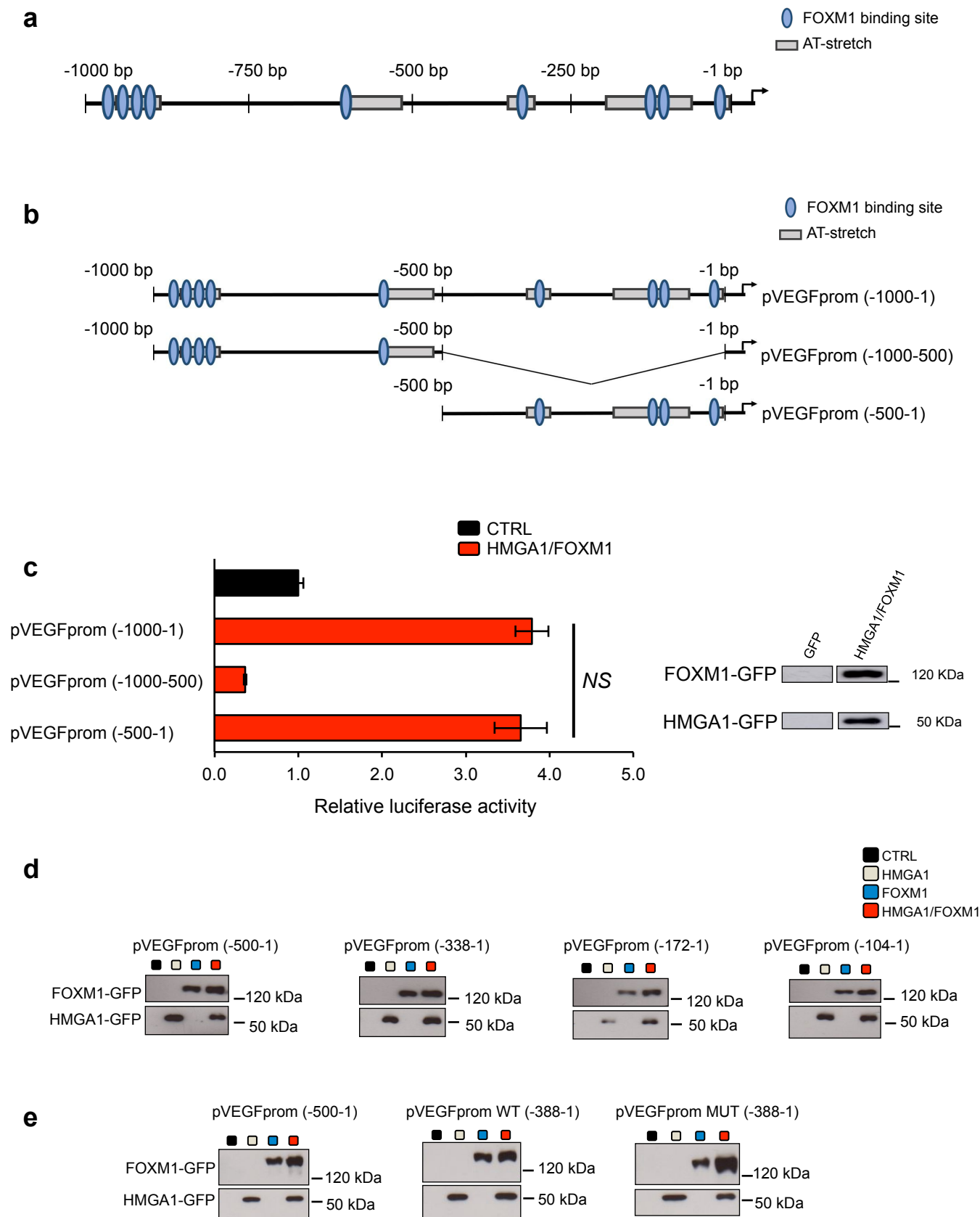

Supplement: Supplementary file 8 — Figure S5. (a) Schematic representation of the bioinformatic analysis of the 1000 bp VEGFA promoter sequence cloned upstream the luciferase sequence in pGL4.10-VEGFprom (− 1000–1) used in reporter experiments. FOXM1 binding sites are represented with light blue ovals, whereas the AT-enriched sequences bound by HMGA1 are figured as grey boxes. In detailed, we found several AT-rich sequences in the region from – 979 to 907 bp, from − 641 to − 521 bp, from − 355 to − 322 bp, from − 169 to − 75 bp, where the AT stretches are particularly long, and from − 17 to − 13 bp from the TSS of the VEGFA promoter. In addition, we found 9 putative FOXM1 binding sites from − 993 to − 922 bp, from − 643 to – 638 bp, from − 326 to − 322 bp, from − 124 to − 104 bp, where it is located the FOXM1 preferential binding sequence TAAACA, and from − 17 to − 13 bp from the TSS of the VEGFA promoter. (b) Schematic representation of deletion reporter vectors pGL4.10-VEGF (− 1000–500) and pGL4.10-VEGF (− 500–1) obtained from pGL4.10-VEGFprom (− 1000–1). (c) Luciferase assay on HEK293T cells transiently co-transfected with the luciferase reporter plasmid pVEGFprom (− 1000–1), the deletion mutants pVEGFprom (− 1000–500) or pVEGFprom (− 500–1) with the expression plasmids pEGFP-HMGA1 and pEGFP-FOXM1. pRL-CMV Renilla luciferase expression vector was included to normalize for transfection efficiencies. Values are reported as relative luciferase activity comparing to cells transfected with the expression plasmid pEGFP. The data are represented as the mean ± SD (n = 3). NS: not significant; two-tailed Student’s t-test. An example of western blot validations is reported. (d) and (e) Representative images of western blot validations of experiments presented in Fig. 5b and d respectively. (PDF 2000 kb) [file 13046_2019_1307_MOESM8_ESM.pdf]

Additional 9: Figure S6.

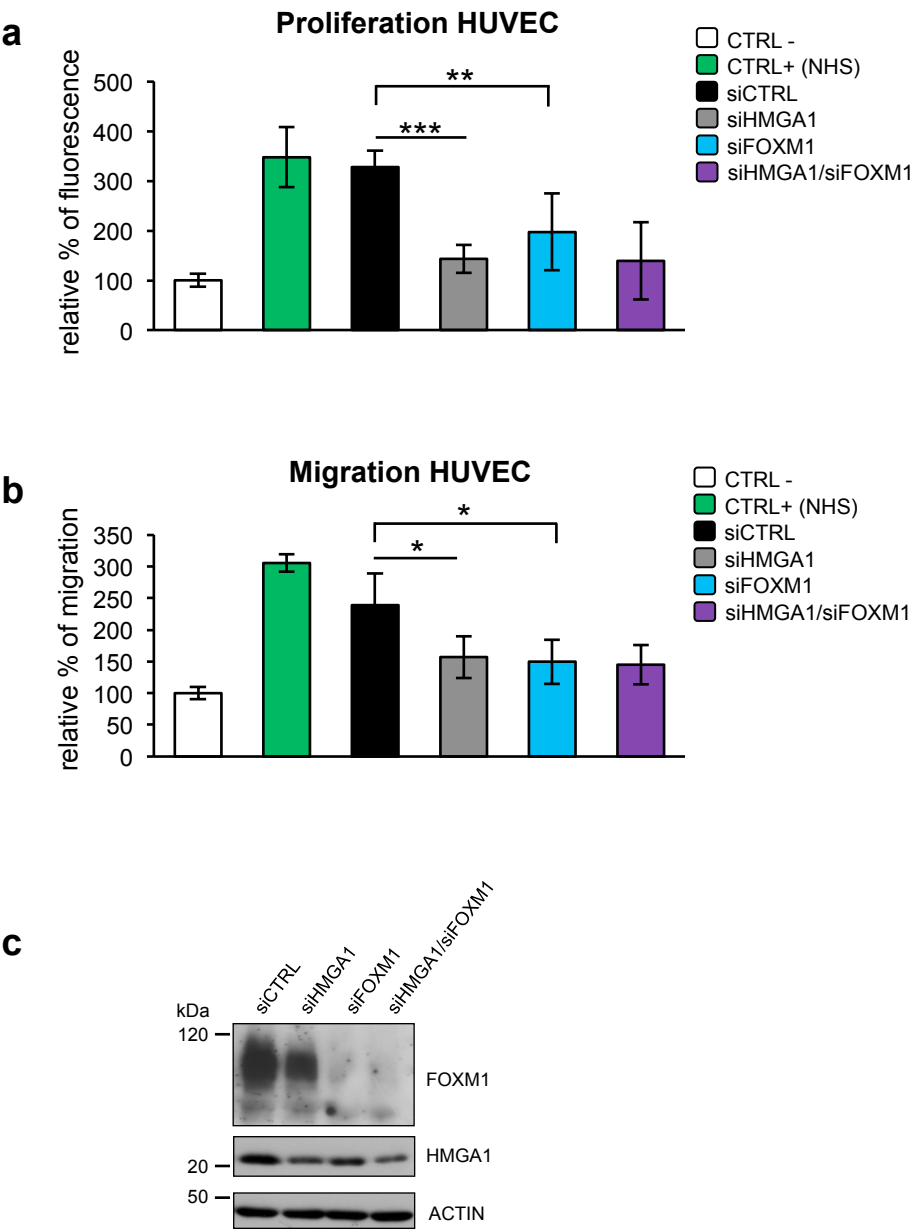

Supplement: Supplementary file 9 — Figure S6. HUVEC cells were treated with MDA-MB-231 cells supernatants, who had been previously silenced for HMGA1, FOXM1 or co-silenced for HMGA1 and FOXM1. Serum-free medium and normal human serum (NHS) were used as negative and positive controls respectively. (a) The proliferation of HUVEC cells was investigated by the positivity to the Ki67 marker and expressed in terms of relative % of fluorescence respect to negative control (CTRL-). The data are represented as the mean ± SD (n > 3); **p < 0.001, ***p < 0.001; two-tailed Student’s t-test. (b) The migration of endothelial cells was assessed by Transwell assay, adding the supernatants of MDA-MB-231 in the lower chambers of the multiwell. The number of cells migrated were counted and the results are expressed as relative % of migration, respect to negative control (CTRL-). The data are represented as the mean ± SD (n > 3). *p < 0.05; two-tailed Student’s t-test. (c) Representative western blot validations of HMGA1/FOXM1 silencing in MDA-MB-231 cells used to collect supernatants are reported. β-actin was used as a loading control. (PDF 704 kb) [file 13046_2019_1307_MOESM9_ESM.pdf]

Additional file 10: Figure S7.

**a**

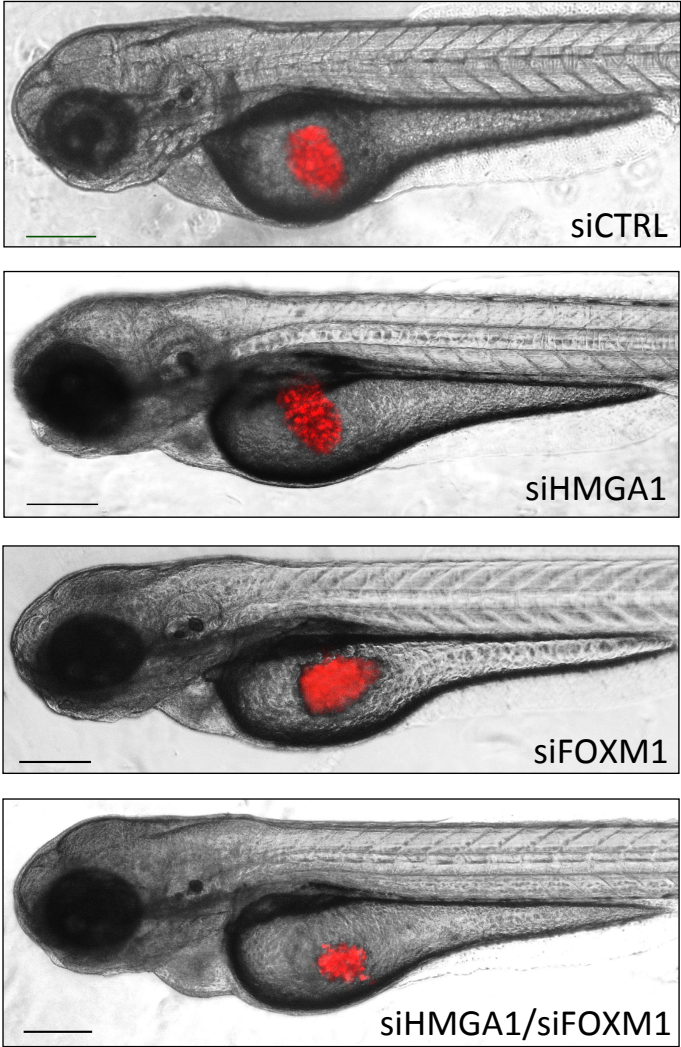

**b**

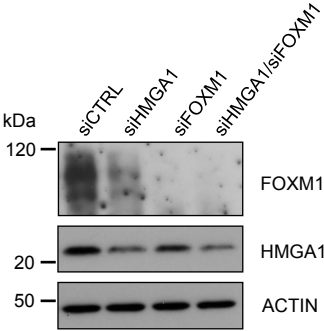

**c**

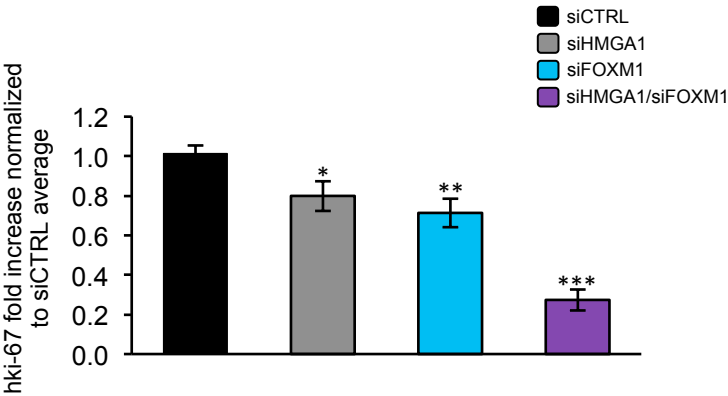

Supplement: Supplementary file 10 — Figure S7. (a) Representative live images of the tumor masses (red) at 1dpi in zebrafish Tg (fli1:EGFP)Y1 embryos microinjected with MDA-MB-231 cells pre-treated with siCTRL, siHMGA1, siFOXM1 or siHMGA1/siFOXM1. Just before the microinjection, tumor cells (red) were stained with the fluorescent DiI stain. Scale bar = 100 μm. (b) A representative western blot validation of HMGA1/FOXM1 silencing in MDA-MB-231 cells microinjected in zebrafish embryos is reported. β-actin was used as a loading control. (c) qRT-PCR analysis of human Ki67 at 1 dpi in control MDA-MB-231 cells (black bar) and MDA-MB-231 cells silenced for HMGA1 (grey bar), FOXM1 (light blue bar) or cosilenced for the two factors (purple bar). Data were normalized to the human GAPDH mRNA amount. The data are represented as the mean ± SD (n = 60). *p < 0.05, **p < 0.01, ***p < 0.001; two-tailed Student’s t-test. (PDF 2803 kb) [file 13046_2019_1307_MOESM10_ESM.pdf]

# Additional file 11: Figure S8.

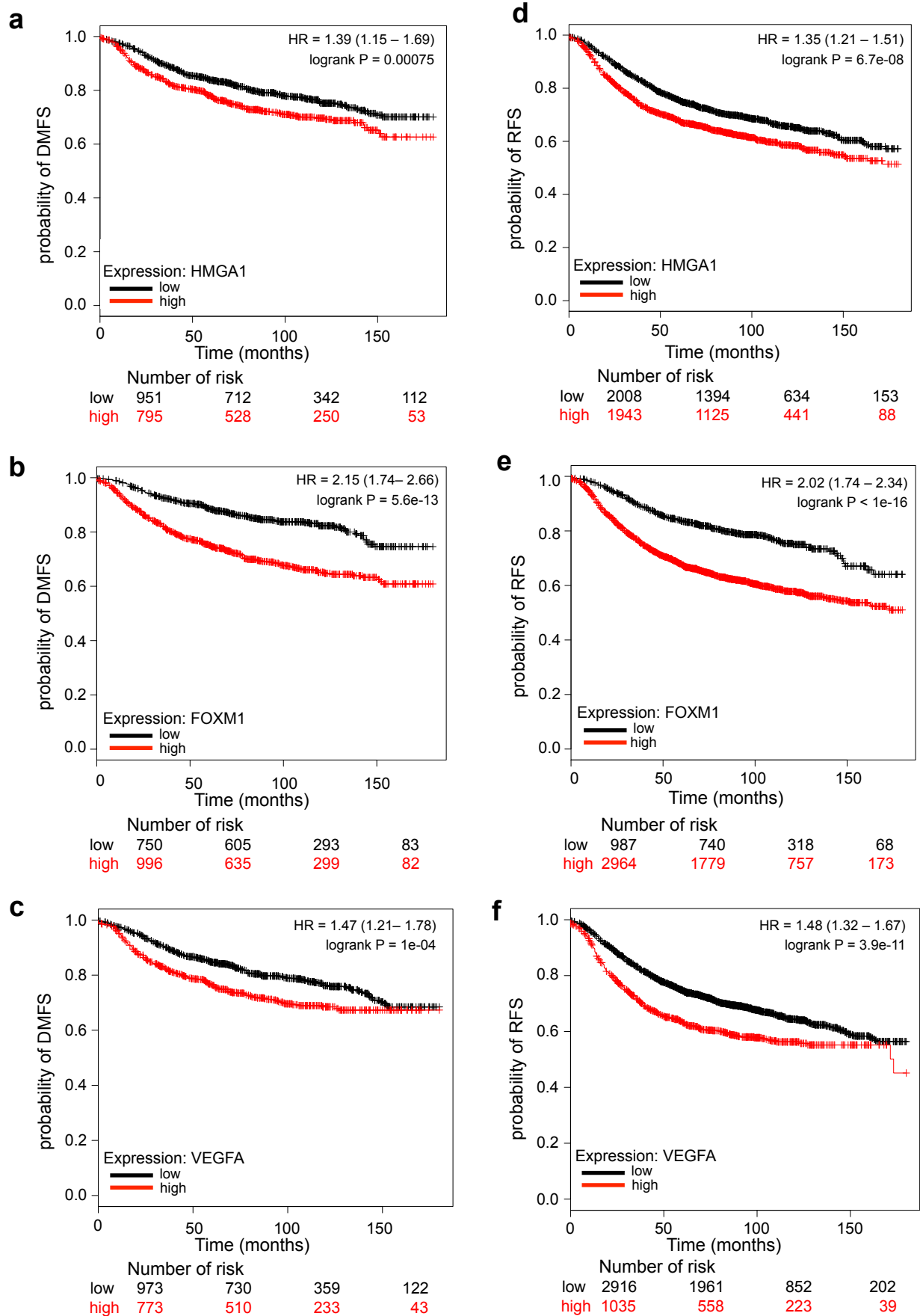

Supplement: Supplementary file 11 — Figure S8. (a-c) Kaplan Meier curves of DMFS in a cohort of breast cancer patients stratified by HMGA1 (a), FOXM1 (b) and VEGFA (c) expression. (d-f) Kaplan Meier curves of RFS in a cohort of breast cancer patients stratified by HMGA1 (d), FOXM1 (e) and VEGFA (f) expression. (PDF 387 kb) [file 13046_2019_1307_MOESM11_ESM.pdf]
