# Supplementary material for: HMGA1 promotes breast cancer angiogenesis supporting the stability, nuclear localization and transcriptional activity of FOXM1
Source: J Exp Clin Cancer Res. 2019 Jul 16;38:313. doi: 10.1186/s13046-019-1307-8 (PMC6636010; doi:10.1186/s13046-019-1307-8)
Supplement: Supplementary file 3 — Tables S2. and S3. GSEA analysis on 24 and 72 h siHMGA1-regulated genes. (PDF 72 kb) [file 13046_2019_1307_MOESM3_ESM.pdf]

Additional file 3: Table S2.

Gene Set Name	Description	FDR q-value
REACTOME_METABOLISM_OF_RNA	Genes involved in Metabolism of RNA	5.5E-51
KEGG_RIBOSOME	Ribosome	5.61E-51
REACTOME_METABOLISM_OF_MRNA	Genes involved in Metabolism of mRNA	2.21E-48
REACTOME_TRANSLATION	Genes involved in Translation	4.15E-42
REACTOME_3_UTR_MEDIATED_TRANSLATIONAL_REGULATION	Genes involved in 3' -UTR-mediated translational regulation	4.68E-42
REACTOME_METABOLISM_OF_PROTEINS	Genes involved in Metabolism of proteins	4.68E-42
REACTOME_INFLUENZA_LIFE_CYCLE	Genes involved in Influenza Life Cycle	2.02E-40
REACTOME_PEPTIDE_CHAIN_ELONGATION	Genes involved in Peptide chain elongation	1.11E-38
REACTOME_NONSENSE_MEDIATED_DECAY_ENHANCED_BY_THE_EXON_JUNCTION_COMPLEX	Genes involved in Nonsense Mediated Decay Enhanced by the Exon Junction Complex	1.17E-38
REACTOME_INFLUENZA_VIRAL_RNA_TRANSCRIPTION_AND_REPLICATION	Genes involved in Influenza Viral RNA Transcription and Replication	1.36E-38
REACTOME_SRP_DEPENDENT_COTRANSLATIONAL_PROTEIN_TARGETING_TO_MEMBRANE	Genes involved in SRP-dependent cotranslational protein targeting to membrane	5.85E-36
REACTOME_IMMUNE_SYSTEM	Genes involved in Immune System	1.26E-33
HALLMARK_OXIDATIVE_PHOSPHORYLATION	Genes encoding proteins involved in oxidative phosphorylation.	6.2E-27
REACTOME_ADAPTIVE_IMMUNE_SYSTEM	Genes involved in Adaptive Immune System	4.25E-25
REACTOME_FORMATION_OF_THE_TERNARY_COMPLEX_AND_SUBSEQUENTLY_THE_43S_COMPLEX	Genes involved in Formation of the ternary complex, and subsequently, the 43S complex	3.52E-24
REACTOME_ACTIVATION_OF_THE_MRNA_UPON_BINDING_OF_THE_CAP_BINDING_COMPLEX_AND_EIFS_AND_SUBSEQUENT_BINDING_TO_43S	Genes involved in Activation of the mRNA upon binding of the cap-binding complex and eIFs, and subsequent binding to 43S	1.53E-23
REACTOME_HIV_INFECTION	Genes involved in HIV Infection	2.18E-23
HALLMARK_MYC_TARGETS_V1	A subgroup of genes regulated by MYC - version 1 (v1).	4.16E-23
HALLMARK_MTORC1_SIGNALING	Genes up-regulated through activation of mTORC1 complex.	3.19E-21
REACTOME_DNA_REPLICATION	Genes involved in DNA Replication	3.79E-19
REACTOME_HOST_INTERACTIONS_OF_HIV_FACTORS	Genes involved in Host Interactions of HIV factors	3.94E-19
REACTOME_CELL_CYCLE_MITOTIC	Genes involved in Cell Cycle, Mitotic	4.27E-19
KEGG_HUNTINGTONS_DISEASE	Huntington's disease	6.92E-19
HALLMARK_P53_PATHWAY	Genes involved in p53 pathways and networks.	1.14E-17
KEGG_ALZHEIMERS_DISEASE	Alzheimer's disease	1.59E-17
REACTOME_MITOTIC_M_M_G1_PHASES	Genes involved in Mitotic M-M/G1 phases	2.78E-17
KEGG_PARKINSONS_DISEASE	Parkinson's disease	4.26E-17
REACTOME_CELL_CYCLE	Genes involved in Cell Cycle	4.53E-17
REACTOME_CELL_CYCLE_CHECKPOINTS	Genes involved in Cell Cycle Checkpoints	4.74E-16
REACTOME_DOWNSTREAM_SIGNALING_EVENTS_OF_B_CELL_RECEPTOR_BCR	Genes involved in Downstream Signaling Events Of B Cell Receptor (BCR)	6.77E-15
REACTOME_APOPTOSIS	Genes involved in Apoptosis	8.43E-15
REACTOME_APC_C_CDC20_MEDIATED_DEGRADATION_OF_MITOTIC_PROTEINS	Genes involved in APC/C:Cdc20 mediated degradation of mitotic proteins	1.09E-14
HALLMARK_DNA_REPAIR	Genes involved in DNA repair.	1.17E-14
REACTOME_TCA_CYCLE_AND_RESPIRATORY_ELECTRON_TRANSPORT	Genes involved in The citric acid (TCA) cycle and respiratory electron transport	1.61E-14
REACTOME_METABOLISM_OF_AMINO_ACIDS_AND_DERIVATIVES	Genes involved in Metabolism of amino acids and derivatives	2.11E-14
REACTOME_CLASS_I_MHC_MEDIATED_ANTIGEN_PROCESSING_PRESENTATION	Genes involved in Class I MHC mediated antigen processing & presentation	2.12E-14
REACTOME_REGULATION_OF_MITOTIC_CELL_CYCLE	Genes involved in Regulation of mitotic cell cycle	2.77E-14
KEGG_OXIDATIVE_PHOSPHORYLATION	Oxidative phosphorylation	3.69E-14
REACTOME_APC_C_CDH1_MEDIATED_DEGRADATION_OF_CDC20_AND_OTHER_APC_C_CDH1_TARGETED_PROTEINS_IN_LATE_MITOSIS_EARLY_G1	Genes involved in APC/C:Cdh1 mediated degradation of Cdc20 and other APC/C:Cdh1 targeted proteins in late mitosis/early G1	8.91E-14
REACTOME_SYNTHESIS_OF_DNA	Genes involved in Synthesis of DNA	1.7E-13
HALLMARK_UNFOLDED_PROTEIN_RESPONSE	Genes up-regulated during unfolded protein response, a cellular stress response related to the endoplasmic reticulum.	2.37E-13
REACTOME_METABOLISM_OF_CARBOHYDRATES	Genes involved in Metabolism of carbohydrates	3.54E-13
REACTOME_SIGNALING_BY_THE_B_CELL_RECEPTOR_BCR	Genes involved in Signaling by the B Cell Receptor (BCR)	3.89E-13
REACTOME_MITOTIC_G1_G1_S_PHASES	Genes involved in Mitotic G1-G1/S phases	3.91E-13
PID_P73PATHWAY	p73 transcription factor network	6.02E-13
REACTOME_ANTIGEN_PROCESSING_UBIQUITINATION_PROTEASOME_DEGRADATION	Genes involved in Antigen processing: Ubiquitination & Proteasome degradation	6.02E-13
REACTOME_S_PHASE	Genes involved in S Phase	8.00E-13
REACTOME_REGULATION_OF_APOPTOSIS	Genes involved in Regulation of Apoptosis	2.11E-12
REACTOME_ANTIGEN_PROCESSING_CROSS_PRESENTATION	Genes involved in Antigen processing-Cross presentation	2.87E-12
HALLMARK_E2F_TARGETS	Genes encoding cell cycle related targets of E2F transcription factors.	3.81E-12

Table S3.

Gene Set Name	Description	FDR q-value
HALLMARK_E2F_TARGETS	Genes encoding cell cycle related targets of E2F transcription factors.	8.05E-87
HALLMARK_G2M_CHECKPOINT	Genes involved in the G2/M checkpoint, as in progression through the cell division cycle.	6.58E-72
REACTOME_CELL_CYCLE	Genes involved in Cell Cycle	1.3E-48
REACTOME_CELL_CYCLE_MITOTIC	Genes involved in Cell Cycle, Mitotic	1.33E-48
REACTOME_DNA_REPLICATION	Genes involved in DNA Replication	1.25E-37
REACTOME_MITOTIC_M_M_G1_PHASES	Genes involved in Mitotic M-M/G1 phases	4.15E-29
HALLMARK_TNFA_SIGNALING_VIA_NFKB	Genes regulated by NF-kB in response to TNF [GeneID=7124].	1.89E-28
KEGG_CELL_CYCLE	Cell cycle	3.17E-24
HALLMARK_EPITHELIAL_MESENCHYMAL_TRANSITION	Genes defining epithelial-mesenchymal transition, as in wound healing, fibrosis and metastasis.	7.54E-24
HALLMARK_ESTROGEN_RESPONSE_EARLY	Genes defining early response to estrogen.	4.99E-23
HALLMARK_MITOTIC_SPINDLE	Genes important for mitotic spindle assembly.	4.99E-23
NABA_MATRISOME	Ensemble of genes encoding extracellular matrix and extracellular matrix-associated proteins	6.49E-23
HALLMARK_GLYCOLYSIS	Genes encoding proteins involved in glycolysis and gluconeogenesis.	3.33E-22
REACTOME_MITOTIC_G1_G1_S_PHASES	Genes involved in Mitotic G1-G1/S phases	3.99E-21
HALLMARK_ESTROGEN_RESPONSE_LATE	Genes defining late response to estrogen.	1.54E-20
HALLMARK_MYC_TARGETS_V1	A subgroup of genes regulated by MYC - version 1 (v1).	1.54E-20
REACTOME_IMMUNE_SYSTEM	Genes involved in Immune System	6.24E-20
PID_E2F_PATHWAY	E2F transcription factor network	2.48E-19
REACTOME_MITOTIC_PROMETAPHASE	Genes involved in Mitotic Prometaphase	3.7E-18
HALLMARK_HYPOXIA	Genes up-regulated in response to low oxygen levels (hypoxia).	4.12E-18
HALLMARK_P53_PATHWAY	Genes involved in p53 pathways and networks.	4.12E-18
REACTOME_DNA_STRAND_ELONGATION	Genes involved in DNA strand elongation	1.07E-17
REACTOME_G1_S_TRANSITION	Genes involved in G1/S Transition	1.1E-17
REACTOME_S_PHASE	Genes involved in S Phase	4.18E-17
HALLMARK_APOPTOSIS	Genes mediating programmed cell death (apoptosis) by activation of caspases.	8.92E-17
NABA_MATRISOME_ASSOCIATED	Ensemble of genes encoding ECM-associated proteins including ECM-affilaited proteins, ECM regulators and secreted factors	1.48E-16
PID_FOXM1_PATHWAY	FOXM1 transcription factor network	6.28E-16
REACTOME_DNA_REPAIR	Genes involved in DNA Repair	8.22E-16
HALLMARK_INFLAMMATORY_RESPONSE	Genes defining inflammatory response.	4.71E-15
HALLMARK_KRAS_SIGNALING_UP	Genes up-regulated by KRAS activation.	4.71E-15
PID_P53_DOWNSTREAM_PATHWAY	Direct p53 effectors	6.47E-15
PID_AURORA_B_PATHWAY	Aurora B signaling	6.47E-15
PID_PLK1_PATHWAY	PLK1 signaling events	1.65E-14
KEGG_PATHWAYS_IN_CANCER	Pathways in cancer	1.85E-14
HALLMARK_IL2_STAT5_SIGNALING	Genes up-regulated by STAT5 in response to IL2 stimulation.	2.31E-14
HALLMARK_MTORC1_SIGNALING	Genes up-regulated through activation of mTORC1 complex.	2.31E-14
REACTOME_SYNTHESIS_OF_DNA	Genes involved in Synthesis of DNA	1.39E-13
REACTOME_E2F_MEDIATED_REGULATION_OF_DNA_REPLICATION	Genes involved in E2F mediated regulation of DNA replication	2.32E-13
HALLMARK_UV_RESPONSE_DN	Genes down-regulated in response to ultraviolet (UV) radiation.	1.19E-12
REACTOME_G2_M_CHECKPOINTS	Genes involved in G2/M Checkpoints	2.39E-12
REACTOME_UNWINDING_OF_DNA	Genes involved in Unwinding of DNA	5.13E-12
KEGG_DNA_REPLICATION	DNA replication	7.62E-12
REACTOME_ACTIVATION_OF_THE_PRE_REPLICATIVE_COMPLEX	Genes involved in Activation of the pre-replicative complex	8.74E-12
KEGG_OOCYTE_MEIOSIS	Oocyte meiosis	2.91E-11
REACTOME_CELL_CYCLE_CHECKPOINTS	Genes involved in Cell Cycle Checkpoints	3.42E-11
REACTOME_DOUBLE_STRAND_BREAK_REPAIR	Genes involved in Double-Strand Break Repair	4.9E-11
HALLMARK_COMPLEMENT	Genes encoding components of the complement system, which is part of the innate immune system.	7.72E-11
REACTOME_CHROMOSOME_MAINTENANCE	Genes involved in Chromosome Maintenance	1.41E-10
PID_P73PATHWAY	p73 transcription factor network	1.69E-10
PID_RB_1PATHWAY	Regulation of retinoblastoma protein	2.64E-10
