# Supplementary material for: HMGA1 promotes breast cancer angiogenesis supporting the stability, nuclear localization and transcriptional activity of FOXM1
Source: J Exp Clin Cancer Res. 2019 Jul 16;38:313. doi: 10.1186/s13046-019-1307-8 (PMC6636010; doi:10.1186/s13046-019-1307-8)
Supplement: Supplementary file 4 — Table S4. List of differentially expressed genes after HMGA1-silencing at 24 (a) and 72 (b) hours in common with FOXM1-gene network. (PDF 35 kb) [file 13046_2019_1307_MOESM4_ESM.pdf]

Additional file 4: Table S4.

a

24 hours

ID	Exp Log Ratio
CAV1	1.158
CENPE	0.801
LEF1	0.758
SNAI2	0.614
CCNE2	0.587
BRIP1	0.548
CCNE1	-0.661

b

72 hours

ID	Exp Log Ratio
NES	1.457
LEF1	1.183
CCNE2	1.132
AURKB	1.061
CENPB	1.059
TOP2A	0.909
BRIP1	0.9
MCM8	0.86
SKP2	0.834
CDK1	0.799
CENPE	0.759
CENPF	0.741
CCNA2	0.74
NEK2	0.627
FOXM1	0.626
ZEB1	0.547
PLK4	0.525
KIF20A	0.513
VEGFA	0.507
LDHA	0.502
CCND2	-0.726
AR	-1.134
VCAN	-1.187
MUC5AC	-1.35
